# Identification of Negative BOLD Responses in Epilepsy Using Windkessel Models

**DOI:** 10.3389/fneur.2021.659081

**Published:** 2021-10-08

**Authors:** Alejandro Suarez, Pedro A. Valdés-Hernández, Byron Bernal, Catalina Dunoyer, Hui Ming Khoo, Jorge Bosch-Bayard, Jorge J. Riera

**Affiliations:** ^1^Neuronal Mass Dynamics Laboratory, Florida International University, Miami, FL, United States; ^2^Nicklaus Children Hospital, Miami, FL, United States; ^3^Montreal Neurological Institute, McGill University, Montreal, QC, Canada; ^4^Department of Neurosurgery, Osaka University, Suita, Japan

**Keywords:** negative BOLD responses, Windkessel models, hemodynamic response function, general linear model, machine learning, epilepsy, EEG-fMRI multimodal

## Abstract

Alongside positive blood oxygenation level–dependent (BOLD) responses associated with interictal epileptic discharges, a variety of negative BOLD responses (NBRs) are typically found in epileptic patients. Previous studies suggest that, in general, up to four mechanisms might underlie the genesis of NBRs in the brain: (i) neuronal disruption of network activity, (ii) altered balance of neurometabolic/vascular couplings, (iii) arterial blood stealing, and (iv) enhanced cortical inhibition. Detecting and classifying these mechanisms from BOLD signals are pivotal for the improvement of the specificity of the electroencephalography–functional magnetic resonance imaging (EEG-fMRI) image modality to identify the seizure-onset zones in refractory local epilepsy. This requires models with physiological interpretation that furnish the understanding of how these mechanisms are fingerprinted by their BOLD responses. Here, we used a Windkessel model with viscoelastic compliance/inductance in combination with dynamic models of both neuronal population activity and tissue/blood O_2_ to classify the hemodynamic response functions (HRFs) linked to the above mechanisms in the irritative zones of epileptic patients. First, we evaluated the most relevant imprints on the BOLD response caused by variations of key model parameters. Second, we demonstrated that a general linear model is enough to accurately represent the four different types of NBRs. Third, we tested the ability of a machine learning classifier, built from a simulated ensemble of HRFs, to predict the mechanism underlying the BOLD signal from irritative zones. Cross-validation indicates that these four mechanisms can be classified from realistic fMRI BOLD signals. To demonstrate proof of concept, we applied our methodology to EEG-fMRI data from five epileptic patients undergoing neurosurgery, suggesting the presence of some of these mechanisms. We concluded that a proper identification and interpretation of NBR mechanisms in epilepsy can be performed by combining general linear models and biophysically inspired models.

## Introduction

The interest in the concurrent electroencephalography–functional magnetic resonance imaging (EEG-fMRI) method as an important imaging modality in epilepsy surgical planning has increased gradually during the last 20 years (1–3). This method generates whole-brain maps of blood oxygenation level–dependent (BOLD) responses evoked by interictal epileptiform discharges (IEDs), which are then used to locate/identify potential irritative zones (IZs) within the brain. IED-evoked BOLD response analysis is attractive for epilepsy surgery planning, owing to its low invasiveness, accessibility, low cost, and efficiency. The standard clinical protocol for using IED-evoked BOLD signal to demarcate IZs includes (a) concurrent EEG and fMRI recordings while the patient is resting or prompted to sleep; (b) IEDs (spikes and sharp waves) are visually identified by EEG technicians; (c) series of IED onsets are convolved with a canonical hemodynamic response function (HRF, 4) to create a set of regressors; (d) the BOLD signal at each voxel is described as a function of these IED-based regressors *via* a general linear model (GLM) (4); and (e) statistical parametric maps (*t*-test and *F*-test) of the linear coefficients are created to detect positive BOLD responses (PBRs). In advanced clinical protocols, IZ detection with the EEG-fMRI technique is performed with flexible parametric HRF models (5–9) to account for voxel and subject variability. Unfortunately, these parametric HRF models misrepresent atypical BOLD responses frequently observed in certain regions of an epileptic brain, e.g., negative BOLD responses (NBRs), precluding detection of the seizure-onset zones (SOZs) in many patients (10–16). Non-parametric HRF models (17–23) could in principle account for HRF misspecification in epilepsy by sacrificing parsimony, but they are computationally intensive, do not include spatial dependencies, and lack mechanistic foundations. These limitations create opportunities to increase the sensitivity of the EEG-fMRI method in epilepsy.

Initial EEG-fMRI clinical studies have associated SOZs with PBRs as a result of a localized hyperemic response triggered by abnormal neuronal excitability [e.g., the pioneer work by (24)]. More recent data suggest NBRs in SOZs might be caused by local circuit inhibitions during after-spike slow-wave components (13), presumably owing to a profound hyperpolarization of pyramidal cells (25, 26) after a fast spike. In general, this is the most accepted mechanism for the NBR found in many experimental paradigms (27–32). The inhibition can occur in the same active region, but exceeding the excitation, provoking negative changes in the overall neuronal activity, resulting in an NBR. For simplicity, we shall refer to these mechanisms as the *enhanced cortical inhibition* (ECI). Clinical [see reviews by (33) and (34)] and preclinical (35) studies of focal epilepsy point out to the existence of abnormal decreases in the hyperemic/metabolic ratio during frequent/strong epileptogenic activity, which might be linked to an NBR according to computer simulations presented in this study. We shall refer to this mechanism as *altered neurometabolic/vascular couplings* (ANCs).

However, not all NBRs might be considered as SOZ candidates during surgical planning. For example, deactivations (or disruption) of normal resting state networks (RSNs), such as the default mode network (DMN), have also been linked to NBRs, a phenomenon reported in epileptic patients during IEDs (11, 36, 37). Here, we refer to this mechanism as *neuronal disruption of network activity* (NDA). Also, an initial work by Harel et al. (38) suggested that *arterial blood stealing* (ABS) could cause an NBR in healthy brain areas as a result of decreases in cerebral blood flow (CBF) and volume (CBV) in a region in close proximity to an IED-evoked PBR. A recent computational model (39) demonstrated that ABS is physically possible in the brain vasculature. More recent studies have corroborated experimentally the existence of ABS (40, 41). Therefore, areas with NDA and ABS types of NBRs are not IZs; hence, they should not be considered during the surgical workup.

Classifying these different types of NBRs from the noise fMRI signal must be challenging. However, it is reasonable to expect they have different HRF waveforms, which could be used as fingerprints of the underlying mechanism. To verify and take advantage of these differences for the identification of the mechanisms, it is advised to have biophysical models. Incorporating biophysical model–based discrimination of disparate NBR types in refractory focal epilepsy may significantly improve the accuracy of the EEG-fMRI method to localize/delineate SOZs, thereby increasing success rates of ablative surgery. Windkessel (balloon) models (42) have been utilized in the last decades for statistical inference of BOLD signals (43, 44) due to their parsimonious capabilities to capture most of the features of the HRFs reported experimentally.

In this article, we propose a comprehensive Windkessel-based model to account for these four possible mechanisms underlying NBRs in patients with focal epilepsy. Using the model, we predict a specific HRF waveform for each of the four NBR mechanisms aforementioned. We also investigate if these HRFs are classifiable from noisy fMRI data. To that end, HRFs were fitted using the near-neighborhood exogenous autoregressive (NN-ARx) (45) model. HRF dimensionality was reduced using the principal component analysis (PCA). We subsequently build a machine learning ensemble classifier that uses the first three principal components as features and their corresponding mechanisms as classes. We evaluate the performance of the classifier in predicting mechanisms from their BOLD signals. Finally, we used this method to evaluate the presence of different NBR types in cases of drug-resistant focal epilepsy.

## Materials and Methods

### EEG-fMRI Data

This is a prospective study duly approved by the Western Institutional Review Board (WIRB #20160218). Parents or legal guardians of 10 patients (9–18 y/o) recruited at Nicklaus Children's Hospital signed a written approved–informed consent. All patients were refractory to pharmacology treatment and exhibited frequent IED. In this context, “frequent” was defined as at least 1 IED per minute. Patients needing sedation or vascular malformations were excluded. In this study, patients exhibiting significant NBRs were only included (*n* = 5). Demographics and clinically relevant findings are summarized in Table 1. The results of EEG source localization, positron emission tomography (PET), ictal single-photon emission computed tomography (SPECT), subdural implantation (ECoG/sEEG), MRI diagnosis, and pathology were annotated. The MR-compatible EEG system used in this study is not Food and Drug Administration–approved. Therefore, results obtained from the EEG-fMRI analysis were not used in any form during the surgical evaluation of the patients.

**Table 1:** Patient clinical information.

**#**	**A/G Eth**	**Seizures**	**IED**	**MRI**	**Nuclear**		**EEG BSI**	**ECoG/sEEG**	**Ictal EEG**	**Surgery**
					***PET***	***Ictal SPECT***				
1	14/F Cauc	Au/To	54	L-H (Pol-MG)	ND	L-FT	L-ST	ND	Monomorph sharps at C3-P3-CZ	NPY
2	13/F Cauc	Ge/UG	38	NL	R-P	R-F	R-FP	R-P	Bi-F spike/SW at 3–4Hz	R-P
3	17/M Cauc	Pa R-FI	48	NL/(PO) R-PI	ND	R-H	R-FO&T	R-FT>> L-T	R-FT>L-T	R-AT-Lo Hi & AI
4	10/M Cauc	Fo R-Le/Pe	81	Tb	ND	ND	ND	NPY	L-F S&W	NPY
5	9/F Hisp	R-Ar/Ting CP/ESES	572	L-C Cyst (vs. Mal)	L-PaC, Hyper	ND	L-PaC	Concordant	L(C3-P3-FZ) S&W	NPY

*Seizures: CP, complex partial; Au, autonomic; To, tonic; Ge, generalized; UG, uncinate gyral; Pa, partial; Fo, focal; Le, leg; Pe, pedaling; Ar, arm; Ting, tingling; Discharges: IED, number of IEDs; SW, slow wave; S&W, spike and wave discharges; Locations: F, frontal; P, parietal; T, temporal; C, central; S(A)T, superior (anterior)–temporal; FP, frontopolar; P(A)I, posterior (anterior)–insular; PaC, paracentral; FT, frontotemporal; FI, frontoinsular; FO, fronto-opercular; Hi, hippocampus. Acronyms: R, right; L, left; H, hemisphere; NL, no lesion; PO, postoperatory; ND, not done; Bi, bilateral; NPY, not performed yet; Lo, lobectomy; Mal, malacia; Pol-MG, polymicrogyria; Tb, tuberous sclerosis; ESES, electric status epilepticus on sleep; A, age; G, gender; Eth, ethnicity; Cauc, Caucasian; Hisp, Hispanic; Symbol: >, effect size*.

We acquired four 10-min trials of simultaneous EEG-fMRI data from each patient. Using the fMRI trials for which the IEDs were better identified from the EEG, we fitted GLM and NN-ARx to estimate IED-related PBRs and NBRs. The IEDs were visually detected and classified into several subtypes by two experts based on their morphology, the semiology of the patient, and other neuroimaging modalities. The different subtypes of IEDs and other types of events were used as different types of inputs (conditions) in GLM and NN-ARx. In addition, motion correction parameters were included as nuisance regressors.

MRI data were collected in a Philips 1.5-T scanner with a 16-channel SENSE Rx coil. fMRI was acquired with a GE-EPI sequence. Each fMRI scan consists of 21 interleaved slices 6 *mm* thick with a 2-mm gap, in-plane voxel size of 3 × 3 *mm*, and field of view (FOV) = 204 mm. Flip angle (FA) was 90, repetition time (*T*_*R*_) = 2 *s*, and echo time (*T*_*E*_) = 45 *ms*. For the purpose of anatomical reference, a high-resolution T1-weighted image was acquired using a spoiled three-dimensional (3D) gradient echo sequence with *T*_*R*_ = 9.7 *ms*, *T*_*E*_ = 4 *ms*, and FA = 12. The structural MRIs have 90 to 100 slices, covering the whole brain. In some cases, a T2-weighted 3D image was also acquired with parameters: *T*_*R*_ = 25 *ms* and *T*_*E*_ = 3.732 *ms*, FA = 30, FOV = 240 mm, and 160 2-mm-thick axial slices. 3D fluid-attenuated inversion recovery volumes were acquired in sagittal plane using the following parameters: *T*_R_ = 4,800 ms, *T*_E_ = shortest; FA = 40°; 230 sagittal cuts, matrix: 212 × 185 × 230 mm; FOV = 250; matrix reconstruction isovoxel 0.98 mm. The fMRI volumes were preprocessed using statistical parametric mapping (SPM) (http://www.fil.ion.ucl.ac.uk/spm/). They were corrected for motion artifacts and spatially smoothed with an 8-mm Gaussian kernel. Both smoothed and unsmoothed images were used in GLM analysis to detect significant voxels. Although the minimum variance estimator of GLM may be biased due to non-Gaussian noise (46), the latter was necessary to detect near PBR and NBR, as it was suggested by Goense et al. (47) and Harel et al. (38) for detecting the NBR of vascular origin. The GLM analysis and the results were masked to the gray matter using the SPM segmentation obtained from the anatomical image (48).

EEG was recorded using a 10–10 system 32-channel EasyCap (BrainAmp MR, Brain Product GmbH). To record EEG data simultaneously with the fMRI signal, we used MRI-compatible EEG amplifiers (BrainAmp MR, Brain Product GmbH). EEG signals were sampled at 5 kHz and digitized (0.5-μV resolution) (BrainVision Recorder 1.4, Brain Products GmbH). The majority of EEG electrodes had impedances lower than 5 kΩ. The electrocardiogram (ECG) was measured with an ECG electrode attached to the middle of the back of the patients. To synchronize the EEG with fMRI scans, a trigger marking the beginning of the scans was sent to the EEG recording laptop. To ensure the highest temporal precision, the clock of the laptop was synchronized to the 10-MHz clock of the MR console using a Syncbox (Brain Products GmbH). The following EEG preprocessing was performed using BrainVision Analyzer 2 (Brain Products GmbH). To remove the MR-related artifact, the EEG data were first subsampled to 50 kHz using *sinc* interpolation to virtually increase its resolution and correct the random phase jittering—of no <0.2-ms resolution determined by the 5-kHz sampling rate—that is present in the scan markers. This phase jittering has significant negative effects in the estimation of the MR gradient artifacts as the latter can change as fast as 0.2 ms. Subsequently, we applied a method for removing the MRI gradient artifacts (49), based on the estimation of an average artifact template. The resultant EEG data were bandpass filtered within 0.5–125 Hz. After marking the R-waves using a semiautomatic tool, we applied a method to detect and remove the effects of the balistocardiogram (50). Finally, we applied ICA based on the Infomax method (51, 52) to remove further artifacts.

As IEDs last ~70–200 ms, the input *u*(*t*) is modeled as a train of short pulses. To account for the actual time in which the slice containing the region with significant voxels was acquired, the inputs *u*(*t*) were transformed according to $u\left(t+n\frac{T_{R}}{N_{z}}\right)$, where *n* is the position of the slice according to the sequence in which they were acquired, and *N*_*z*_, the number of slices the whole scan.

Ictal SPECT was performed using a Siemens Multispect 3 gamma camera (Hoffman States, IL). Technetium-HMPAO was used as the radiotracer at a dose of 300 μCi/kg with a minimum dose of 3 mCi and a maximum dose of 20 mCi. PET was performed using a GE Discovery-Dimension ST PET/CT system. FDG was injected at a dose of 140 μCi/kg with a minimum of 1 mCi and maximum of 15 mCi. The stereo-EEG was performed utilizing a Natus Neurology, Natus Medical Incorporated, Excel-Tech Ltd (XLTEK) (Ontario, Canada). The electrode type was a Natus Neuro Grass disposable deep cup electrode (silver chloride, AgAgCl).

### The Biophysical Model

The proposed model comprises a two-state dynamic causal modeling component (P-DCM) (53) for principal excitatory cells and inhibitory interneurons, extended to having long-range modulatory excitatory inputs also in the inhibitory population. NDA and ECI types of NBRs are explained by adjusting the time constants of the modulatory synaptic connections in the excitatory and inhibitory population of the extended P-DCM model, respectively. Each brain region has a Windkessel component linked to its neuronal activity through an inducing signaling (i.e., the neurovascular coupling). A viscoelastic non-linear delayed compliance was also included (54). To account for blood stealing effects in brain regions sharing a common supply artery (Option 1, Supplementary Table A1), an inductive element was added to connect their respective Windkessel components (39). For regions not sharing a common supplying artery, the two equations for the CBF become independent. For this particular case, a simplified model proposed by Friston et al. (55) is used (Option 2, Supplementary Table A1). An oxygen-to-tissue transport (OTT) component was used to account for the dynamics of the oxygen extraction fraction and the O_2_ concentrations in both tissue and blood (i.e., the neurometabolic coupling) (56). ABS and ANC types of NBRs are explained by fitting those parameters in the model controlling the stealing effect size and the vascular/metabolic imbalance, respectively. The differential equations describing the biophysical model are shown in Supplementary Table A1. Supplementary Figure A1 shows the flow diagram for the physiological mechanisms with their respective state variables (Supplementary Table A2). A graphical representation of all model configurations and defining parameters for each of the four mechanisms is shown in Supplementary Figure A2. Values of the parameters for all these particular situations are summarized in Supplementary Table A3. All the specifics related to the particular cases of the model can be found in the Appendix.

### Detecting IZs Using the GLM

The fastest and most widely used method to detect significant BOLD responses is the GLM regression (4):

(1) $$\begin{array}{r} y\left(t_{i}\right)=\overset{N_{u}}{\underset{k=1}{\sum }}(\beta_{hrf}^{\left(k\right)}\left(h⊗u_{k}\right)\left(t_{i}\right)+\beta_{der}^{\left(k\right)}\left(h\prime ⊗u_{k}\right)\left(t_{i}\right)\\ \;+\beta_{disp}^{\left(k\right)}\left(d⊗u_{k}\right)\left(t_{i}\right))+\overset{N_{r}}{\underset{r=1}{\sum }}\beta_{r}x_{r}\left(t_{i}\right)+\eta \left(t_{i}\right) \end{array}$$

where {*t*_*i*_}_*i* = 1, …, *N*_, being *N* the number of scans and *N*_*u*_ the number of types of inputs {*u*_*k*_(*t*)}_*k* = 1, …, *N*_*u*__, {*x*_*r*_}_*r* = 1, …, *N*_*r*__ the confounding or nuisance regressors (e.g., the motion parameters), and β_*r*_ their effect sizes. *h*, *h*′, and *d* are the canonical BOLD HRF (57), its temporal derivative, and its dispersion, respectively. The noise is prewhitened by applying a first-order autoregressive (AR) model to the signal. To demonstrate that the NBR mechanisms are detectable using GLM, we simulated a set of 10-min BOLD signals with a single type of input (*N*_*u*_ = 1) consisting of a random Poisson train of pulses with average frequency of 2.6/min. This input was the same across all models and trials. We then created a simulated set of *N* = 300 fMRI scans with *T*_*R*_ = 2 *s* by adding the simulated BOLD signals to the voxels of a real EPI image. The trials from the same type of model were added to neighboring voxels to form spatial clusters. The amplitude of the simulated BOLD signal in each cluster was multiplied by a Gaussian spatial kernel [full width at half maximum (FWHM) = 2.5 mm] with the maximum at the center of the cluster. The resultant set of images was further corrupted with colored noise, according to a spectral density given by 1/*f*^*p*^, with 0 < *p* < 1, to account for biological noise inherent to the BOLD signal but not attributable to the temporal filtering of the HRF (58). No nuisance regressor was included in these simulations, i.e., *x*_*r*_ = 0. Following standard fMRI preprocessing procedures, the simulated fMRI scans were spatially smoothed with a Gaussian kernel of 8-mm FWHM. For each voxel, the vectors of coefficient $\beta ={\left[\begin{array}{c} \beta_{can}\&\beta_{der}\&\beta_{disp} \end{array}\right]}^{T}$ of the GLM were estimated using SPM (4). Using an *F* contrast, we selected the voxels where the null hypothesis, **I**_3_*β* = **0** was rejected with *p* < 0.05, after correcting for multiple comparisons using the family-wise error criterion, i.e., the significant voxels. **I**_3_ is the 3 × 3 identity matrix, and ⊗ denotes temporal convolution.

### Estimation of HRFs for the IZs

Although the responses fitted by the GLM, i.e., the first-order Volterra kernel (59), can reconstruct a large variety of HRFs, we used a more adaptable method to accommodate all possible extreme HRF waveforms, the NN-ARx (45), which has been simplified here to extract the HRFs from fMRI time series from the detected IZs. In this particular case, there is no need for a term describing the near-neighbor effect. Under this assumption, the NN-ARx consists of an AR model with an exogenous input *s*_*i*_, i.e., the IEDs.

(2) $$\begin{array}{r} y_{i}=\mu_{i}+\overset{p}{\underset{j=1}{\sum }}\varphi_{j}y_{i-j}+\overset{r}{\underset{j=1}{\sum }}\theta_{j}s_{i-j-d}+\varepsilon_{i} \end{array}$$

With *y*_*i*_ = *y*(*t*_*i*_) defining the BOLD signal at discrete time instants. The term μ_*i*_ accounts for any drift in the BOLD signal, which is modeled by a polynomial. ε_*i*_ represents a normally distribute noise with zero mean and variance σ to be estimated from the fMRI data. This model is suitable to deal with the colored nature of the fMRI time series and the spatial correlation between neighboring voxels in the images. A simple recursive method to estimate the linear coefficient φ_*j*_ and θ_*j*_ can be found in Riera et al. (45). By minimizing the Akaike information criterion, NN-ARx estimates the orders and coefficients of the AR (*p*), the order of a polynomial modeling the drift, and the delay of the HRF onset (*d*). With these, the HRF can be constructed (45). By applying the NN-ARx to the unsmoothed fMRI scans, we extracted the HRF in the IZs with significant PBR and NBR from the GLM.

### Classification of Mechanisms

Once the IZs are detected and their HRF estimated using the NN-ARx method, it is necessary to classify them according to the type of NBR mechanism. For this purpose, we propose to build a classifier based on a machine learning algorithm. We simulated *M* = 51 trials of 10-min fMRI signals with *T*_*R*_ = 2 *s* with random inputs consisting of trains of short pulses Poisson-distributed in time. To account for the entire span of HRF waveforms, we model the possible intraindividual and interindividual variability in the parameters by randomly sampling their values, for each trial, from a uniform distribution within the intervals specified in Supplementary Table A3. For all trials, the NN-ARx–based HRFs were normalized by the maximum of their absolute value. The number of time points of the HRFs was $\frac{T}{T_{R}}=\frac{32}{2}=16$. The PCA was used to reduce the dimension of each HRF to just its three most relevant components (features). Then, a final matrix 5*M* × 3 of features was created, with number 5 representing the number of classes (i.e., ECI, NDA, ANC, and ABS). The PBR class was included as reference. This matrix of features and the corresponding vector of classes were used to create a multiclass machine learning ensemble classifier based on support vector machine (SVM) (60, 61). This type of algorithm is a very popular and powerful tool for classification and regression in many of the research fields today (62). We tested the ability of the classifier to differentiate among the five different classes trying with different kernel functions to find the optimal classification.

## Results

### Predicted Responses for Single Impulses

**Figure 1:**
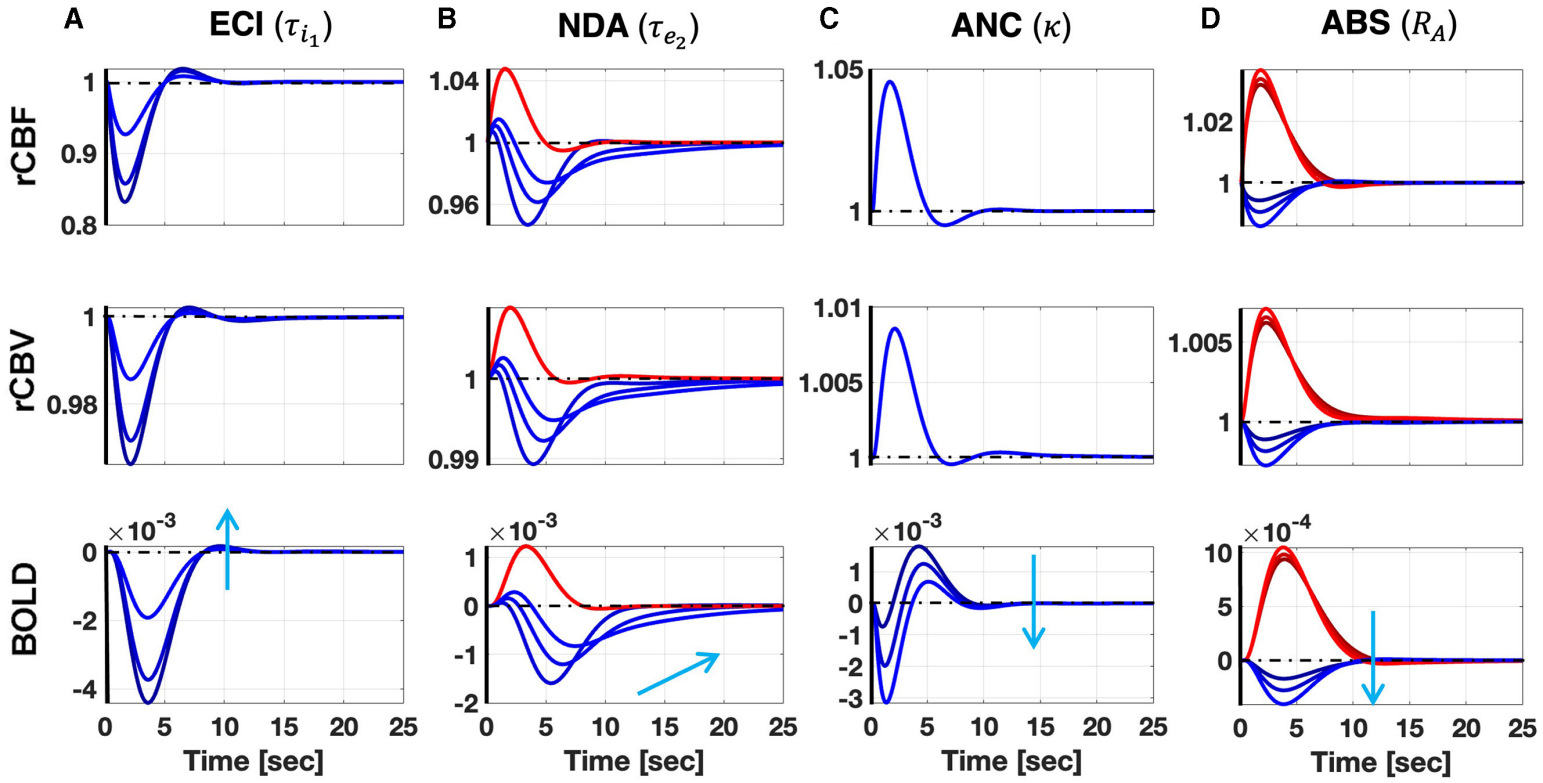
Simulation of the ECI **(A)**, NDA **(B)**, ANC **(C)**, and ABS **(D)** mechanisms after a single short pulse. Red (blue) corresponds to PBR (NBR). Each column corresponds to variation in a parameter that significantly determines the NBR waveform. The sensibility of the responses to these parameters is illustrated with different curves corresponding to three different values of the parameters covering the ranges in Supplementary Table A3. The light blue arrow indicates how the NBRs change by increasing the value of the parameters. Besides BOLD responses, we also show other candidate observables in MRI: rCBF and rCBV. Increasing the duration of the inhibitory recovery decreases the amplitude of the ECI mechanism. Expectedly, the longer the recovery time constant in the NDA mechanism, the slower the NBR. We also note that higher neurometabolic coupling gain yields higher NBR amplitudes in the ANC mechanism. Besides, the higher the arterial resistance, relative to the arteriole, in the ABS mechanism, respectively, the higher NBR amplitude.

Figure 1 shows the predicted responses for the mechanisms proposed in this article, and their sensibility to the relevant parameters, following a single IED event. A longer inhibitory recovery creates an ECI type of NBR with smaller amplitude (Figure 1A). The recovery time of the network is reflected in the NBR duration of NDA (Figure 1B). For ANC, a disproportionately high neurometabolic to neurovascular coupling ratio yields NBR (Figure 1C). Regarding the vascular phenomena, the NBR only occurs in the presence of blood resistance in the shared vessel. The higher the resistance, the higher the amplitude of the NBR (39) (Figure 1D). As Supplementary Material, we uploaded the model codes to the public. The folder includes a pdf file with the documentation that contains user instructions (http://web.eng.fiu.edu/jrieradi/NBR-Model/).

### Detection, Estimation, and Classification of NBR Mechanisms

**Figure 2:**
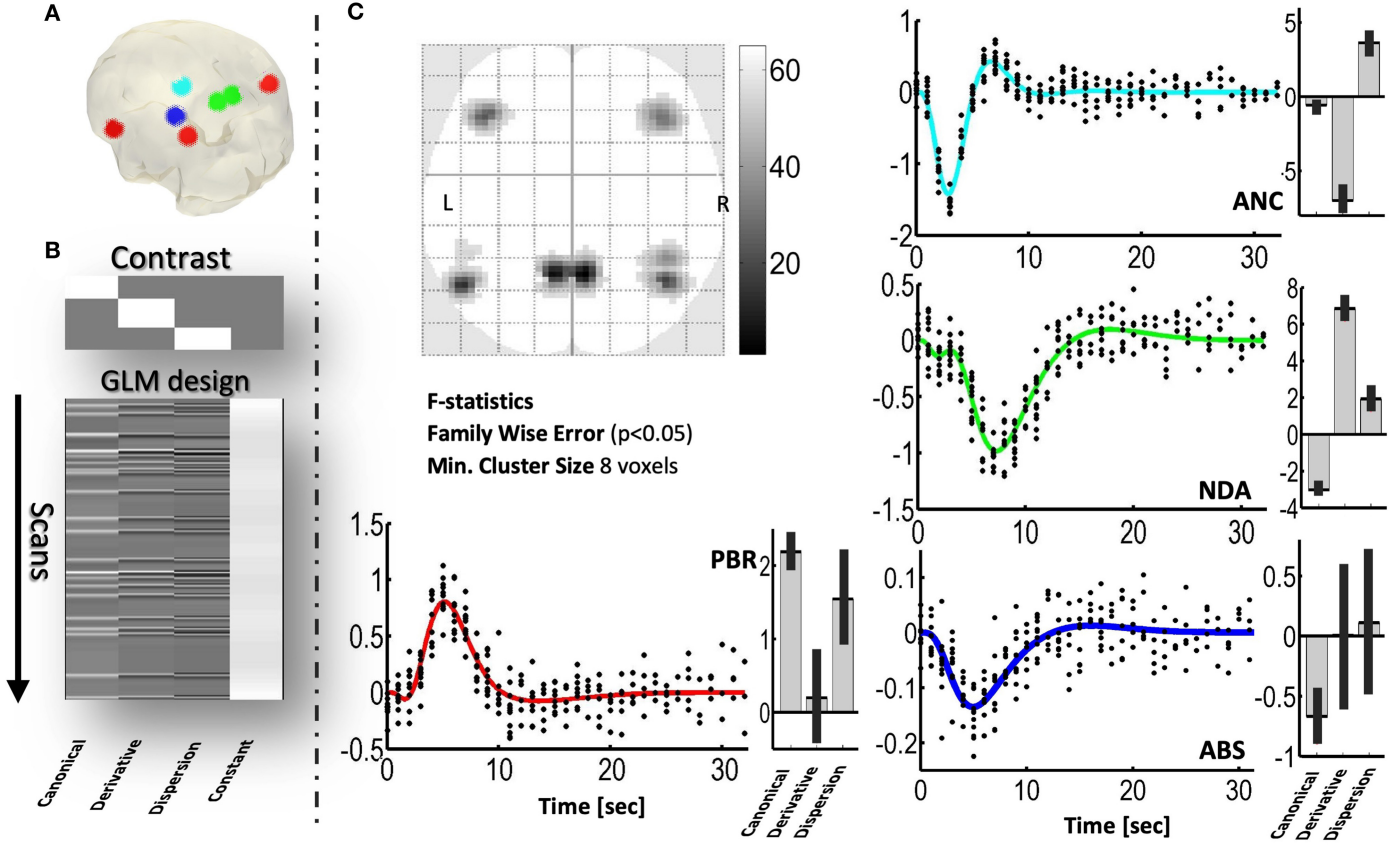
Detection, using the GLM, of simulated spatial distribution of PBRs and three NBRs in a realistic sequence of echoplanar fMRI scans. **(A)** Three-dimensional glass brain showing the regions where the mechanisms were simulated. **(B)** F contrast (the 3-order identity matrix) and design matrix of the GLM, used to detect significant voxels. **(C)** Two-dimensional glass brain showing the *F* statistics. The plots show the responses predicted by the GLM (color curves) and the adjusted data (black dots), for each mechanism. Besides each plot, the estimated values and confidence intervals of the coefficients, β_*can*_, β_*der*_, and β_*disp*_, of the GLM are shown. PBR (red), NDA (green), ANC (cyan), ABS (blue).

Figure 2 indicates that by using the GLM it is possible to detect voxels exhibiting different simulated NBR types. The mechanism with the least significance is ABS—because its NBR has the smaller amplitude. The ECI mechanism was not included because of the similarity of the HRF with that of the ABS mechanism.

**Figure 3:**
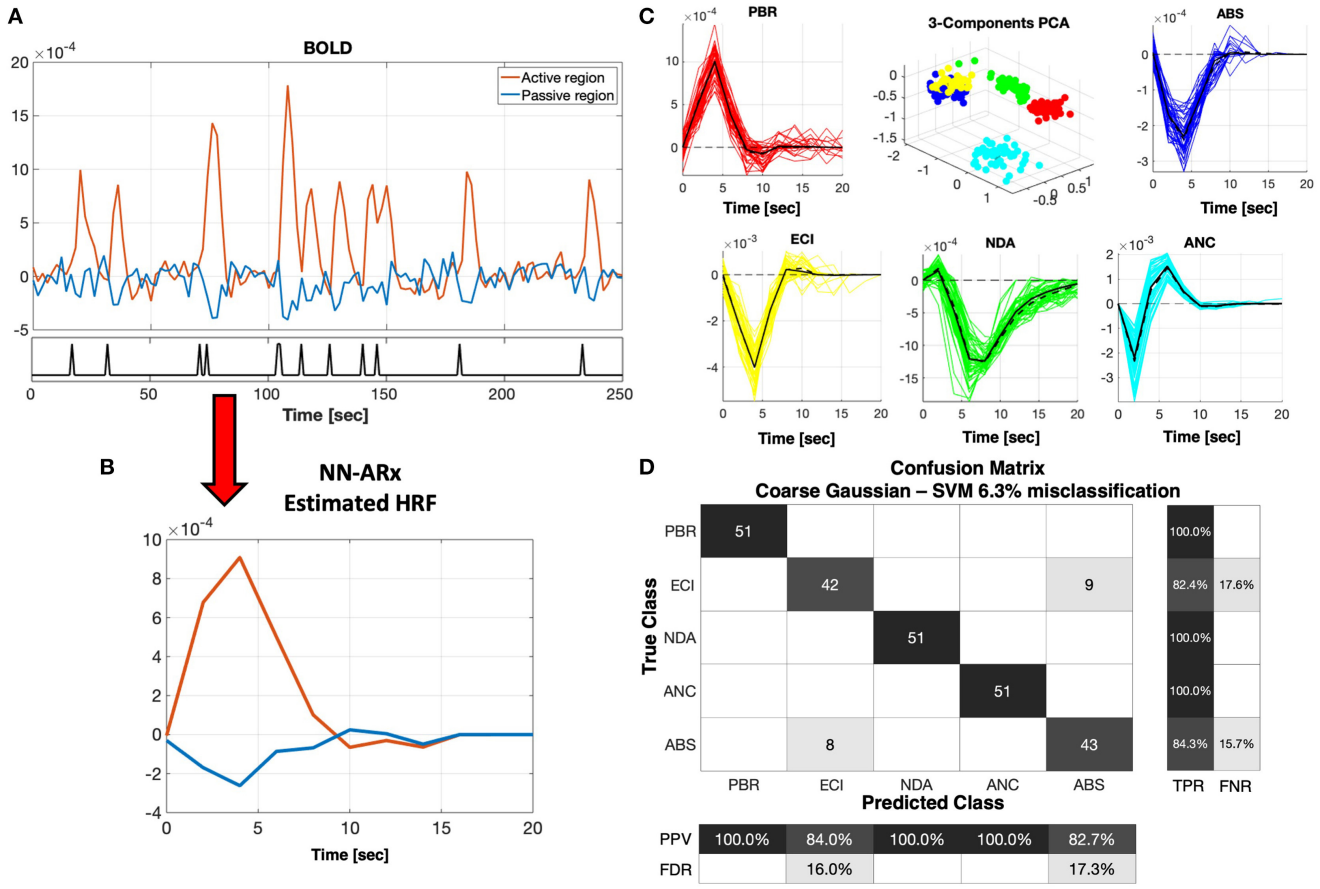
NN-ARx estimation and classification of HRFs. **(A)** Example of a portion of simulated fMRI time series for the ABS mechanism. The input *u*(*t*) is represented with a black trace at the bottom graph. **(B)** Both positive and negative HRFs estimated using NN-ARx from the example in A. **(C)** Estimated HRFs from all trials. The black continuous curve represents the average across trials, whereas the black dash curve corresponds to a simulation without noise. To geometrically illustrate their separability, the 3D plot depicts the scores of the first three components of the PCA decomposition of the matrix formed by stacking all HRF as row vectors, after normalizing their amplitudes. PBR (red), ECI (yellow), NDA (green), ANC (cyan), and ABS (blue). **(D)** Results of the five-fold cross-validation of the SVM classifier with a coarse Gaussian kernel function resulted in 6.3% of misclassification. Center figure shows the confusion matrix. TPR, true-positive rate; FNR, false-negative rate; PPV, positive predictive value; FDR, false discovery rate. As expected, the PBR is distinguishable from all the NBRs. NDA and ANC mechanisms were also perfectly classified, whereas ECI and ABS are the ones that are closer to each other.

Figure 3 shows the performance of the machine learning classifier based on the SVM analysis of ensembles of simulated HRFs as described in section Materials and Methods. We demonstrate the ability of this classifier to predict new mechanisms using a five-fold cross-validation, i.e., leaving five HRFs out for prediction and using the rest as the training set. Six kernel functions were tested (i.e., linear, quadratic, cubic, fine Gaussian, medium Gaussian, and coarse Gaussian). However, we present here only the results obtained with the coarse Gaussian kernels, which provided the best range of accuracy from a minimum 89% to a maximum 93.7%. Trivially, the PBR response is clearly separable from the NBRs. The ANC is distinguishable from the PBRs, even though its HRF can have a significant positive overshoot. NDA and ANC can be distinguished from each other and from the rest of the NBR types, owing to the prolonged recovery of the former and the fast and bipolar shape of the latter. However, the margin of classification and the confidence of prediction of ECI and ABS are the lowest because of their proximity. This means that it might be difficult to distinguish in some cases, at least merely from fMRI signals. In general, mechanisms were incorrectly classified in ~6.3% of the cases, only among ECI and ABS types.

### NBR Mechanisms Associated With Particular IZs in the Epileptic Patients

In this section, we used the HRF classifier, previously trained with data from the biophysical models, to predict the NBR types in some particular IZs of five patients with refractory focal epilepsy. For each case, we applied the following pipeline: (a) GLM-based detection of voxels significantly correlated with the IEDs (SPM); (b) selection of the region-of-interest (ROI) for the IZ of interest; (c) estimation of the NN-ARx HRF, averaged inside spheres within the ROIs; (d) classification of the mechanisms using the machine learning (SVM) classifier; and (e) estimation of key parameters of the biophysical model associated with the identified mechanism. To obtain confidence intervals for the HRFs, we estimated the empirical distribution of the null hypothesis of no significant response using a permutation test. This was done by estimating the NN-ARx HRFs from 5,000 trials with random order of the IEDs. For each time point, the lower and upper confidence values were the 5 and 95 percentiles of these null HRF distributions, respectively. Not all IZs detected for each patient by the EEG-fMRI technique are discussed in this study. Results from these five patients are introduced only as proof of concept. This part of the study does not aim at clinically validating our methodology, but rather at illustrating its value.

**Figure 4:**
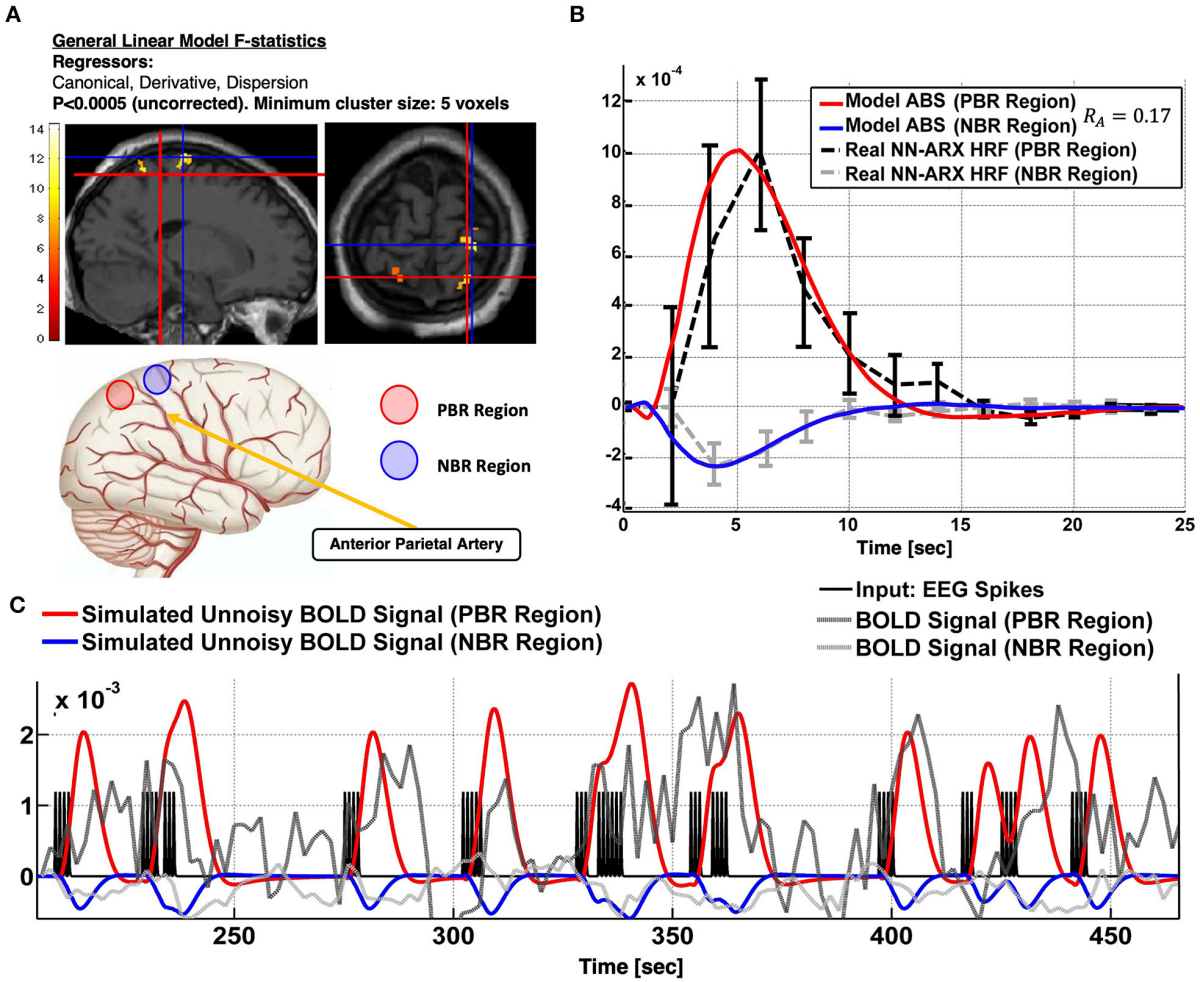
Patient #1 shows an irritative zone with ABS-type HRF. **(A)** Slices showing the thresholded F-statistics map built from the estimated coefficients of the GLM overlaid on the T1-weighted image. The blue crosshair locates the center of the NBR region in the Right Post-central Gyrus; whereas the red crosshair locates the center of the PBR region in the Right Superior Parietal Lobule (SPL). The cartoon at the bottom illustrates the ABS mechanism-the regions share the final segment of the Anterior Parietal Artery. **(B)** The dark gray curve shows the estimated NN-ARx PBR-HRF and its confidence interval, estimated from the real data, and averaged across the voxels within a 10 mm-radius sphere with origin in red crosshair in **(A)**. The light gray curve shows the estimated NN-ARx NBR-HRF and its confidence interval, estimated from the real data, and averaged across the voxels within a 10 mm-radius sphere with origin in the blue crosshair in **(A)**. The light red and blue curves show the unnoisy simulated PBR-HRF, NBR-HRF of the fitted ABS model with the estimated values R_A = 0.17. **(C)** Temporal behavior of the ABS simulated BOLD in the PBR and NBR regions (with the above-mentioned estimated parameters), the time series of the real fMRI and the input used in the NN-ARx estimation.

Patient 1 is a 14-year-old girl with partial autonomic evolving to tonic seizures and left hemisphere polymicrogyria. We found a PBR–NBR pair surrounding the anterior parietal artery (Figure 4). Slices showing the thresholded *F* statistics map built from the estimated coefficients of the GLM using SPM overlaid on the T1-weighted image are presented in panel A. The blue crosshair locates the center of the NBR region in the right postcentral gyrus (PG), whereas the red crosshair locates the center of the PBR region in the right superior parietal lobule (SPL), separated by the postcentral sulcus (another PBR in the left SPL is also shown in this panel). The schematic to the bottom illustrates the ABS mechanism—the regions share the final segment of the anterior parietal artery. The dark gray curve in panel B shows the NN-ARx PBR-HRF and its confidence interval, estimated from the real data, and averaged across the voxels satisfying *F* ≥ 4.5 within a 10-mm radius sphere with origin in red crosshair in panel A. The light gray curve shows the estimated NN-ARx NBR-HRF and its confidence interval and averaged across the voxels satisfying *F* ≥ 5 within a 10-mm radius sphere with origin in the blue crosshair in panel A. The light red and blue curves show the unnoisy simulated PBR-HRF and NBR-HRF of the fitted ABS model with the estimated value *R*_*A*_ = 0.17. In panel C, the temporal behaviors of the ABS simulated BOLD in the PBR and NBR regions (with the aforementioned estimated parameters) overlap the time series of the real fMRI and the 54 IEDs (input) used in the NN-ARx estimation. Note that, to detect this NBR/PBR pair, the unsmoothed images had to be used, considerably decreasing statistical significance. This is however the strategy used in Goense et al. (47) and Harel et al. (38) to detect close BOLD responses with inverted polarities. According to the predicted HRF type, this NBR should not be classified as an IZ.

**Figure 5:**
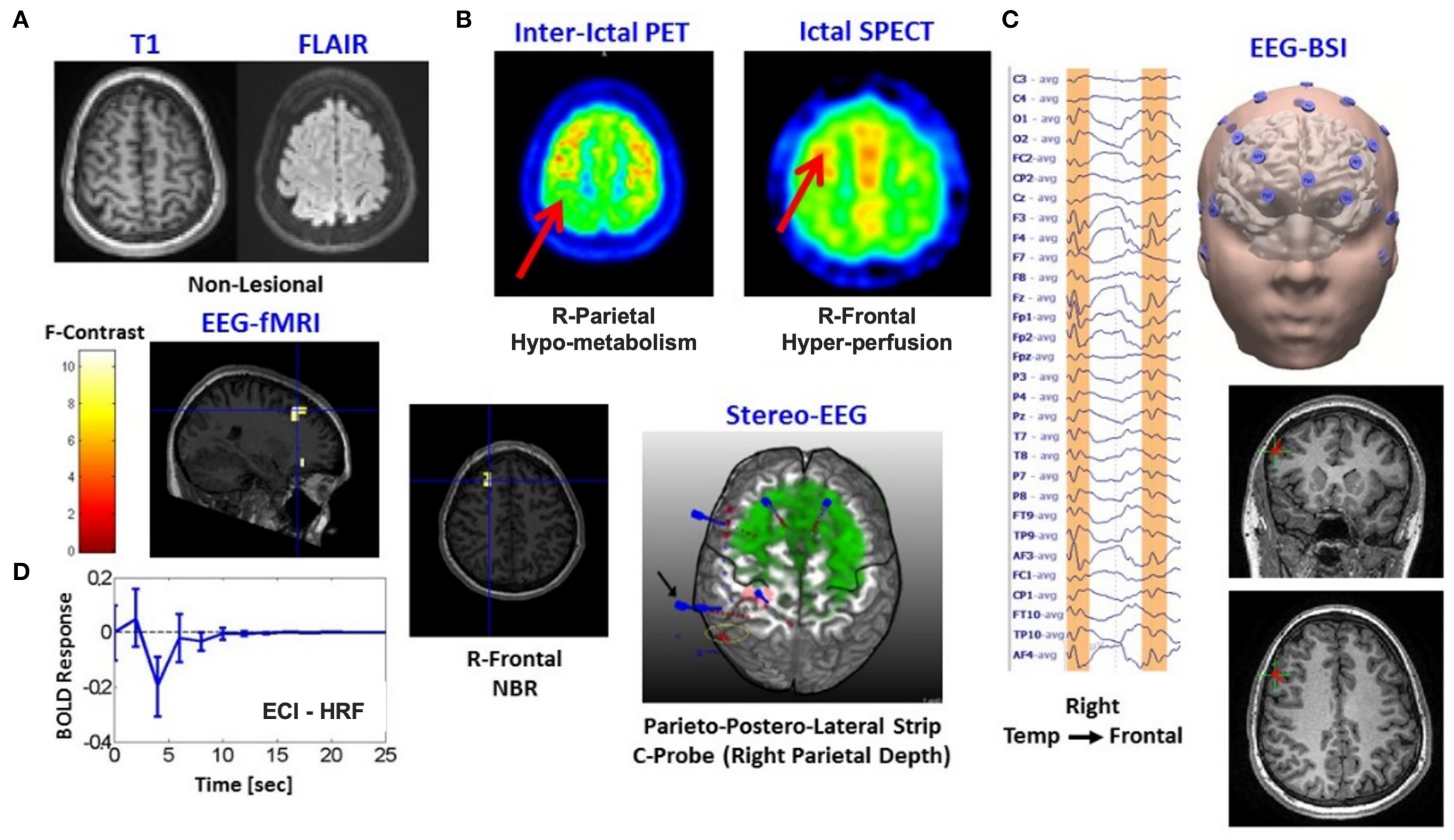
Patients #2 shows an irritative zone with ECI-type HRF with a high probability to be the seizure onset zone. **(A)** Two modalities of fMRI anatomical imaging: left, T1-weighted; right, FLAIR. **(B)** Two modalities of nuclear imaging: left, interictal PET; right, ictal SPECT. **(C)** Left, Ictal EEG points out to a bifrontal spike/slow wave at 3-4Hz; right, brain source imaging (EEG-BSI) in Right Tempo-Frontal. **(D)** Composed panel: left top and center, two slices with the thresholded F-statistics map built from the estimated coefficients of the GLM overlaid on the T1-weighted image with blue crosshair locating the center of one NBR region in the Right Frontal Lobe, left bottom-estimated NN-ARx of this particular NBR-HRF was classified as ECI mechanism, right-stereo EEG (sEEG) electrodes placement is shown as reference.

Patient 2 is a 13-year-old girl with generalized and uncinate gyral seizures. An NBR with ECI type of HRF was found in this patient (Figure 5). No lesions were present (A). In concordance with the ictal-SPECT (hyperperfusion, B) and the brain source imaging (EEG-BSI) (C), we found an NBR in the right frontal eye field. sEEG data (C) showed better correspondence with a hypometabolism (interictal PET, B) in the right parietal lobe. Ictal EEG points out to a bifrontal spike/slow wave at 3 to 4 Hz. Our classifier linked this particular NBR-HRF to an ECI mechanism (D). A total of 38 IEDs were used to generate the BOLD signal regressors. The right parietal lobe was removed. The patient was free of seizures for 30 weeks. Thermal ablation of the right posterior cingulate gyrus was performed 7 months later obtaining a reduction of 90% of seizure frequency. According to our hypothesis, the right frontal eye field is an IZ with potential to be the SOZ; hence, seizures could resume. An alternative explanation is that the ECI might be reflecting the presence of an inhibitory mechanism linked to the ictal slow-wave component.

**Figure 6:**
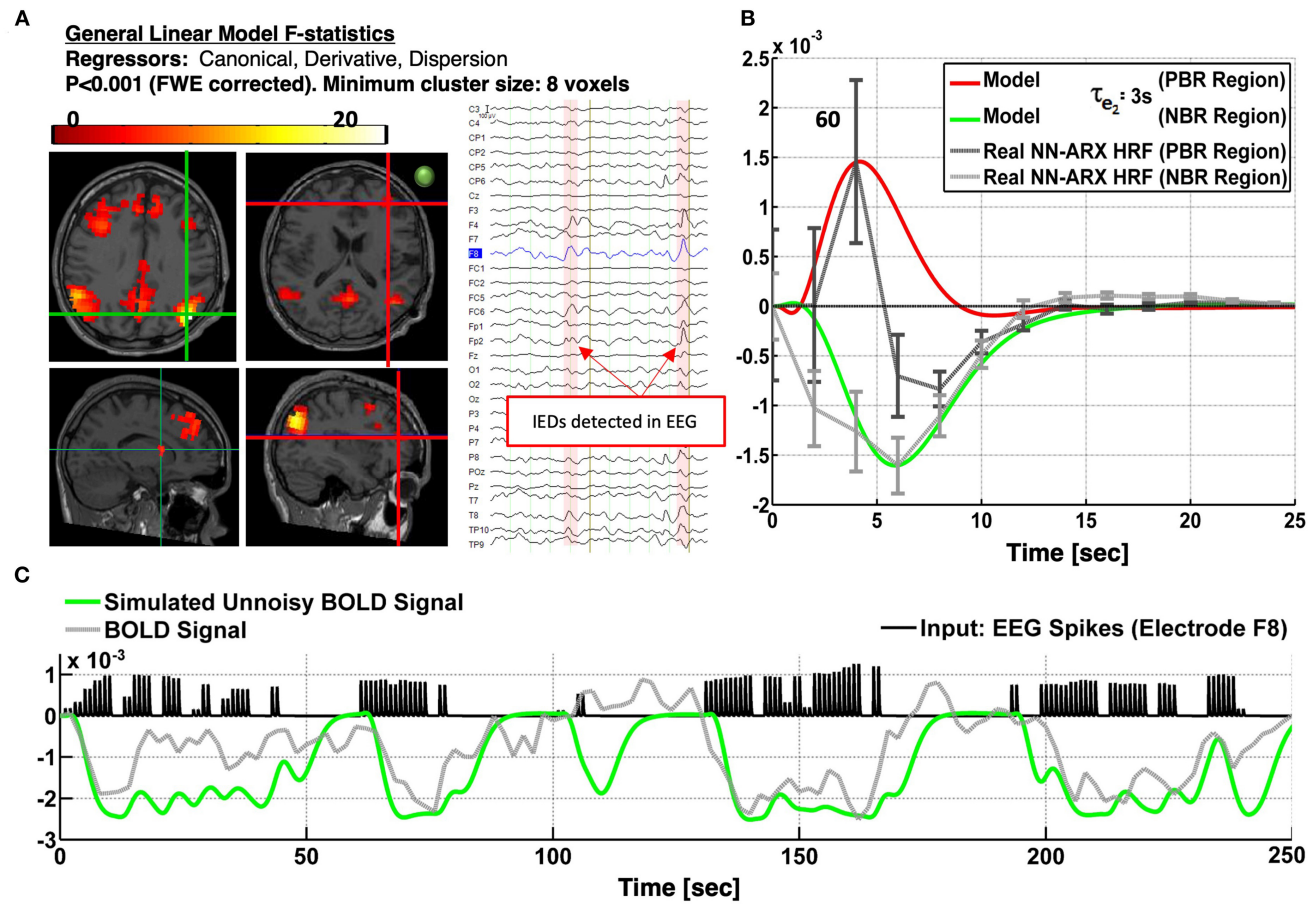
Patients #3 shows an irritative zone with NDA-type HRF in a brain area of the DMN. **(A)** Slices showing the thresholded F-statistics map built from the estimated coefficients of the GLM overlaid on the T1-weighted image. Top left axial slice: green crosshair locating the center of one NBR region in the Right Lateral Parietal node of the DMN—coinciding with the maximum value of the F-statistics. Bottom left sagittal: green crosshair locating another NBR region in the caudate nucleus. Right slices: Red crosshair locating the center of the PBR region in the right frontal cortex-presumably in the origin of the IEDs. The IEDs were detected using the EEG signal in electrode F8. The approximate location of this electrode is shown with a green circle in the right axial slice to illustrate the possible relation of frontal PBR and the IEDs. The right inset shows a short segment of the preprocessed EEG data where 2 IEDs were identified. **(B)** The dark gray curve shows the estimated NN-ARx PBR-HRF and its confidence interval, estimated from the real data around the region marked by the red crosshair in **(A)**. The light gray curve shows the estimated NN-ARx NBR-HRF and its confidence interval, estimated from the real data around the DMN node marked by the green crosshair in the top left slice in **(A)**. The red and green curves show the unnoisy simulated PBR-HRF and NBR-HRF, respectively, for a τ_*e*_2__ = 3*s*. **(C)** For illustration purposes, we also show the temporal behavior of the simulated neuronal activity and BOLD signal in the NBR region [with τ_*e*_2__ = 3*s*], the time series of the real fMRI and the input. To account for the actual relative effect of the IEDs, the amplitude of the input pulses was multiplied by the normalized power of the EEG in F8.

Patient 3 is a 17-year-old boy with partial seizures in the right frontal insula. There are unarguably several regions with NDA type of NBRs linked mainly to the DMN (Figure 6). All nodes of the DMN, as well as the superior frontal gyri, the middle frontal gyri, the left inferior frontal gyrus (IFG), part of the right IFG, the right fronto-opercular region, and the right caudate nucleus, were highly significantly deactivated. In addition, PBR was detected in the right IFG, which could be one of the foci of the IEDs, based on the semiology of the patient and their proximity to the EEG electrodes used to detect the IEDs (i.e., spikes with highest amplitude in electrode F8). Other types of events were also marked and used as regressors in the linear models. Panel A shows the thresholded *F* statistics map built from the estimated coefficients of the GLM overlaid on the T1-weighted image. Top left axial slice: green crosshair locating the center of one NBR region in the right lateral parietal node of the DMN—coinciding with the maximum value of the *F* statistics (bottom left sagittal: green crosshair locating another NBR region in the caudate nucleus; right slices: red crosshair locating the center of the PBR region in the right frontal cortex—presumably in the origin of the IEDs). The approximate location of electrode F8 is shown with a green circle in the right axial slice to illustrate the possible relation of the frontal PBR and the IEDs. The right inset shows a short segment of the preprocessed EEG data where 2 IEDs were identified. In panel B, the dark gray curve shows the estimated NN-ARx PBR-HRF and its confidence interval, estimated from the real data around the region marked by the red crosshair in panel A and averaged across voxels satisfying *F* ≥ 9.5 within a 7-mm radius sphere. The light gray curve shows the estimated NN-ARx NBR-HRF and its confidence interval, estimated from the real data around the DMN node marked by the green crosshair in the top left slice in in panel A and averaged across the voxels satisfying *F* ≥ 70 within a 10-mm radius sphere. The red and green curves show the unnoisy simulated PBR-HRF and NBR-HRF, respectively, of the fitted model with the estimated value of the recovery time constant: τ_*e*_2__ = 3*s*. For illustration purposes (panel C), we also show the temporal behavior of the simulated BOLD signal in the NBR region (with τ_*e*_2__ = 3*s*), the time series of the real BOLD signal, and the IEDs (input). To account for the actual relative effect of the IEDs, the amplitude of the input pulses was multiplied by the normalized power of the EEG in F8. The patient underwent a right anterior temporal lobectomy and partial hippocampus/anterior–insular resection. The patient is not yet seizure-free.

**Figure 7:**
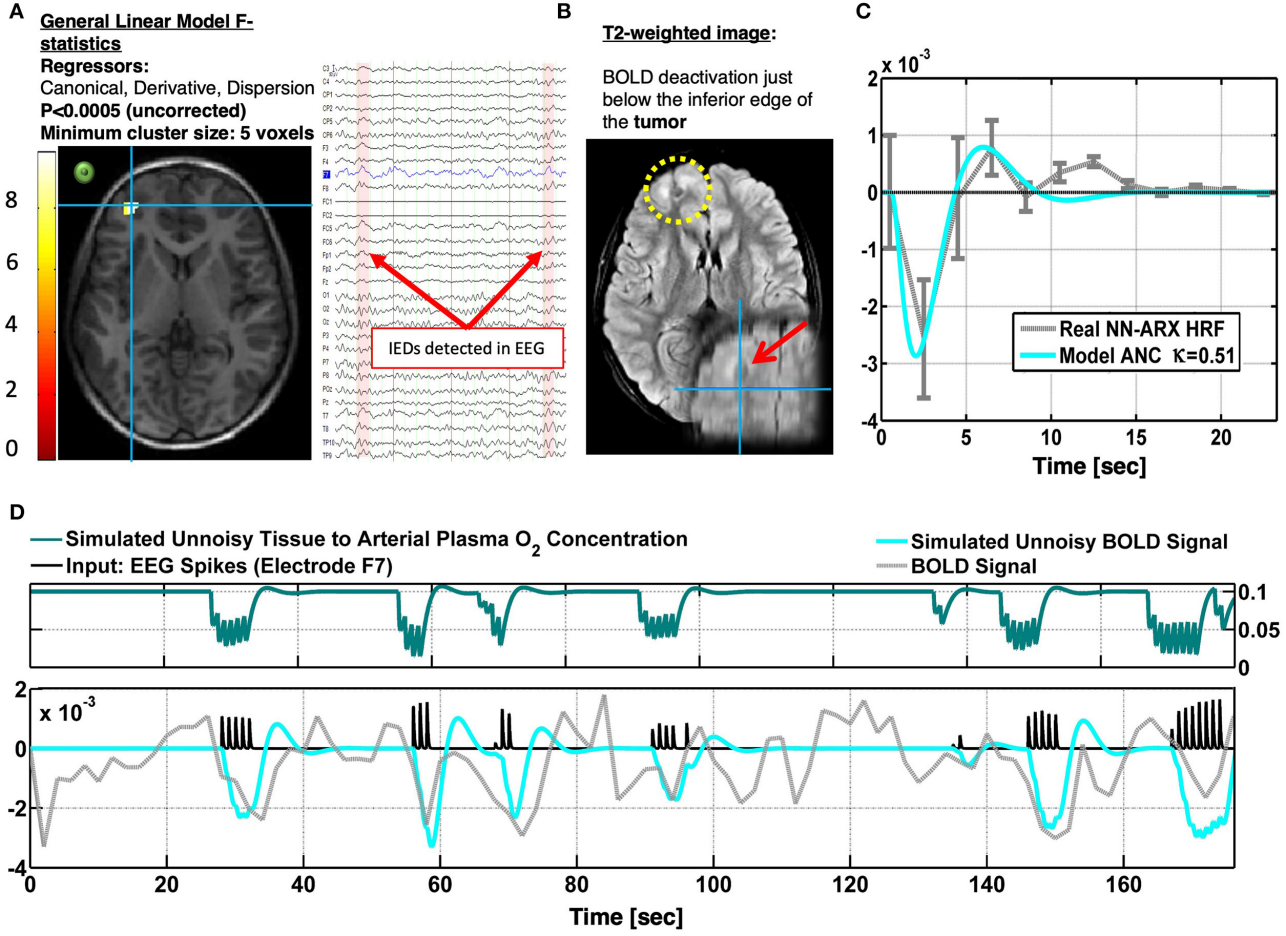
Patients #4 shows an irritative zone with ANC-type HRF in a brain area at the edge of a tumor (TSC). **(A)** Axial slice of the thresholded F-statistics map built from the estimated coefficients of the GLM overlaid on the T1-weighted image. The IEDs were detected using the EEG signal in electrode F7. The approximate location of this electrode is shown with a green circle to illustrate the possible relation between the NBR region and the IEDs. The right inset shows a short segment of the preprocessed EEG data where 2 IEDs were identified. **(B)** The patient suffers from tuberous sclerosis complex. The cyan crosshair—the maximum value of the F-statistics—locates the center of the NBR region, in the perimeter and below one of the patient's tumors. The tumor is highlighted with the yellow circle in the axial slice and the red arrow in the coronal slice of the T2-weighted image. **(C)** The gray curve shows the estimated NN-ARx HRF and its confidence interval, estimated from the real data, and averaged across the voxels within a 10 mm-radius sphere with origin in the crosshair in **(A)**. The cyan curve shows the unnoisy simulated HRF of the fitted OTT model with the estimated value of the neuro-metabolic coupling gain: κ = 0.51 *s*^−1^. **(D)** For illustration purposes, we also show the simulated temporal behavior of the state variable g and the simulated BOLD in the NBR region [with κ = 0.51 *s*^−1^], the time series of the real fMRI and the input. To account for the actual relative effect of the IEDs, the amplitude of the input pulses was multiplied by the normalized power of the EEG in F7.

Patient 4 is a 10-year-old boy with focal seizures and leg pedaling. The patient exhibits ANC-type NBR (cyan crosshair), just in the edge of a tumor in the left frontal cortex (Figure 7). Although the null hypothesis in the significant voxels could not be rejected with a probability corrected by multiple comparisons, this probability was set to a very low value (*p* < 0.0005), and the minimum cluster size of the significant regions was set to five voxels (by decreasing the minimum size of significant voxels, more significant voxels appear in the upper edge of the lesion). Moreover, the HRF was significant according to the permutation test. This HRF corresponded to 50 IEDs identified in electrode F7. Other IEDs, for a total of 81, were also identified and included as regressors in the linear models. Panel A shows an axial slice of the thresholded *F* statistics map built from the estimated coefficients of the GLM overlying on the T1-weighted image. The IEDs were detected using the EEG signal in electrode F7 (green circle), which was very close to the area with the ANC type of NBR. The right inset shows a short segment of the preprocessed EEG data where 2 of 50 IEDs were identified. The patient suffers from tuberous sclerosis complex (B). The cyan crosshair—the maximum value of the *F* statistics—locates the center of the NBR region, in the perimeter, and below one of the patient's tumors. The tumor is highlighted with the yellow circle in the axial slice and the red arrow in the coronal slice of the T2-weighted image. In panel C, the gray curve shows the estimated NN-ARx HRF and its confidence interval, estimated from the real data, and averaged across the voxels satisfying *F* ≥ 6.5 within a 10-mm radius sphere with origin in the crosshair in panel A. The cyan curve shows the unnoisy simulated HRF of the fitted OTT model with the estimated value of the neurometabolic coupling gain: κ = 0.51 *s*^−1^. We also show the simulated temporal behavior of *g* and the simulated BOLD signal in the NBR region (with κ = 0.51 *s*^−1^), the time series of the real fMRI, and the IEDs (input). Also, to account for the actual relative effect of the IEDs, the amplitude of the input pulses was multiplied by the normalized power of the EEG in F7. Our EEG-fMRI results predict the SOZ in the periphery of the tumor. The patient has neither been sEEG implanted nor undergone a surgical procedure.

**Figure 8:**
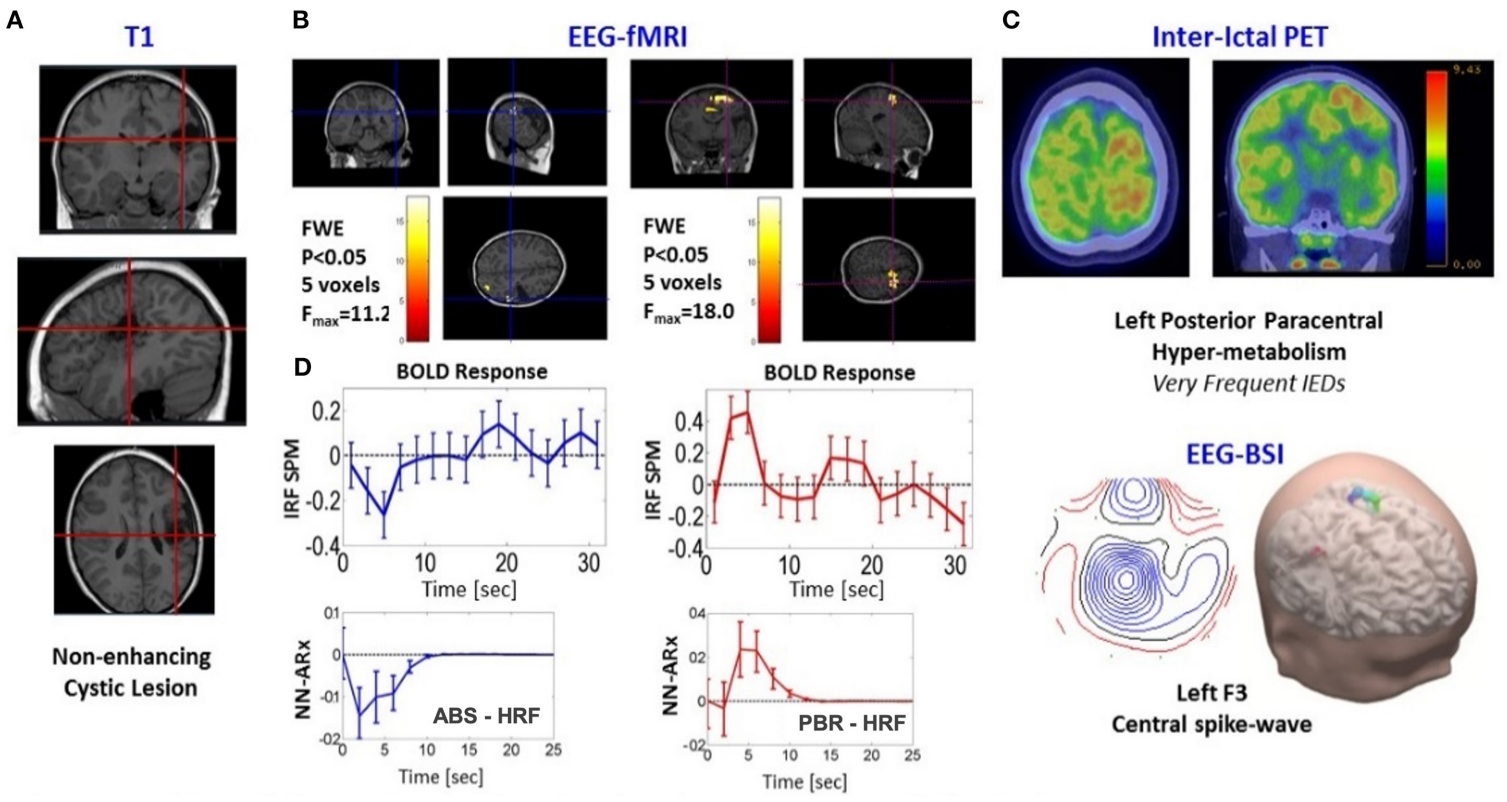
Patients #5 shows an irritative zone with ABS-type HRF. **(A)** Three different slices of anatomical fMRI imaging (T1-weighted) show non-enhancing cystic lesion in the Left Posterior Frontal Lobe (red crosshairs). **(B)** Slices showing the thresholded F-statistics map built from the estimated coefficients of the GLM overlaid on the T1-weighted image; left panel, NBR highlighted with blue crosshairs; right panel, PBR highlighted with red crosshairs. **(C)** Composed panel: top, two slices of nuclear imaging (interictal PET); bottom, EEG-BSI data. **(D)** Comparison between the NN-ARx method with the impulse response function (IRF) method (SPM software). The IRF method was not able to capture underlying HRFs. Results from the NN-ARx were classified as the ABS mechanism.

Patient 5 is a 9-year-old girl with electric status epilepticus on sleep and left (C3-P3-FZ) ictal spikes–waves discharges. A very significant PBR was found in the premotor cortex with high probability to be the SOZ (Figure 8). There is a non-enhancing cystic lesion in the left posterior frontal lobe (A). Interictal PET (C) reveals hypermetabolism (perhaps due to the high frequency of IEDs) on the left posterior paracentral (both precentral and postcentral sulcus) in agreement with the PBR. EEG-BSI indicates brain sources on the bank of the left central sulcus (C). Hence, we expect total seizure control if this area is resected. In contrast, we found an NBR posterior to the cyst that was classified as ABS. We found a PBR nearby this deactivation, but it was not significant. To illustrate the usefulness of the NN-ARx method, we compared HRFs estimated with it and those obtained with the impulse response function (IRF) method (SPM software). The IRF method was not able to capture underlying HRFs. Results from the EEG-fMRI analysis are shown in panel D.

## Discussion

The most accessible, inexpensive, and least-invasive brain imaging method available for localizing seizure foci with high sensitivity (85%) is fMRI combined with EEG. Unfortunately, its benefits remain controversial for many patients, owing to an incomplete understanding of the neuronal, vascular, and metabolic responses in epileptic tissues, decreasing the sensitivity of the biomarker used to locate foci. A large percentage (~17.4%) of discordance in the use of the EEG-fMRI technique is due to a poor classification of clinically relevant NBR responses (Table 2). Here, we hypothesize that NBRs during IEDs could be caused by both clinically and non-clinically relevant mechanisms. A clinically relevant mechanism should be that resulting as a direct consequence of epileptogenic tissues, i.e., an enhanced inhibition and a vascular/metabolic balance mismatch both due to tissue overexcitability. A secondary effect, such as blood flow stealing and resting-state network shutdown, should be considered not clinically relevant and hence not discussed during the surgical workup. Therefore, tools aiming at the classification of these four mechanisms might increase accuracy in the localization of foci for neurosurgical excision, improving success rates. Henceforth, we discuss the rationale and implications of the mechanisms proposed for NBR genesis in epilepsy.

**Table 2:** Results from two studies (different laboratories) about the accuracy of the EEG-fMRI technique, with specifications to (a) typical percentage of refractory epileptic patients who will not be able to complete a successful EEG-fMRI study and (b) typical percentage of these patients with a “discordant” NBR result.

**References**	**No**.	**Removed** ***[Art, No IED]***	**Concordance (%)**			**Discordant (%)**			**NBR-/Tot *(%)***
			***PBR***	***NBR***	***Total***	***PBR***	***NBR***	***Total***	
Salek-Haddadi et al. (14)	63	29 (46.0%)	17	4	62%	2	9	32%	9/34 (26%)
An et al. (1)	47	12 (25.5%)	21	6	77%	5	3	23%	3/35 (8%)
**Total**	110	41 (37.3%)	38	10	69%	7	12	27%	12/69 (17.4)

### Modeling NBRs With a Neuronal Network Origin

Inhibition-related phenomena, i.e., ECI and NDA, require accounting for the imbalance between the local activation of inhibitory and excitatory neuronal states, which depend on their respective connectivity structure. A two-state model (P-DCM) was used by Havlicek et al. (44, 53) to explain NBR during static and flickering visual stimulation. In this article, we extended the P-DCM model to include an additional external input to the inhibitory population in each brain region of interest and an IED-evoked synaptic modulation of the RSNs. Long-range excitatory (thalamocortical/corticocortical) inputs targeting inhibitory populations in the granular layers of the cerebral cortex have been extensively reported in previous literature. IEDs are mostly initiated by a brief increase in excitatory feedback gains and decrease in the thresholds for firing (63). In many cases, local neuronal excitability is followed by an enhancement in cortical inhibition (e.g., the wave component in the spike-wave events), which has been linked to a robust hyperpolarization in III/V layer pyramidal cells (25, 26). Data by Pittau et al. (13) suggested this type of enhanced inhibition might cause NBR in, or near, the actual IZs. Therefore, we hypothesize NBR with an ECI-HRF type should be included as a potential candidate for ablation in the epilepsy surgical workup. We believe the NBR results from an abnormal enhancement in the external input to inhibitory populations in the neocortex, which is modeled by a large response time of the inhibitory population τ_*i*_1__. In this article, the τ_*i*_1__ parameter was fitted using the BOLD data to accurately represent the particular NBR waveform. On the other hand, the recovery of the neuronal activity of the disrupted RSN (NDA) after an IED affects one of its nodes is characterized by a modulation of the excitatory synapses in specific areas within the RSNs, which was characterized by a reduced intralaminar excitatory connectivity *c*_*e*_2__ = 0.01 and a large response time τ_*e*_2__. The latter actually depends on the way the different nodes interact to effectively “shut down” and recover the network. Note that brain dynamics operate near criticality (64–66), i.e., on the brink to instability. Neuronal activities in this situation require higher recovery time to reach equilibria after perturbed and are associated with large-scale dependencies and scale invariance (67). Therefore, the time response for excitatory τ_*e*_2__ was fitted to the BOLD data to accurately characterize the NDA type of NBRs.

### Modeling NBRs With a Vascular/Metabolic Origin

Enhancements in the neurovascular coupling gain ε cause increases in CBF, hence a larger PBR effect. Ictal hyperperfusion has been observed with ^15^O-H_2_O PET and ^99m^Tc-HMPAO/ECD (33, 34). Using invasive recordings from a preclinical model of epilepsy, we have reported increases in the perfusion gain around the SOZs (35). In this previous study, we associated a small value of κ in the SOZ with an increase in the baseline O_2_ metabolism, which might be related to reported glucose hypermetabolism in the SOZs from ictal FDG PET. The interplay between O_2_/glucose metabolism and blood perfusion during IEDs is still controversial. Several studies have shown a hypometabolism in IZs, whereas others have reported complex glucose metabolism patterns with hypermetabolism also in some SOZ candidates (34, 68). Using ^15^O-H_2_O PET, Bittar et al. (68) showed an increase in blood perfusion during IEDs. However, reductions in perfusion have been also reported in the past (34). If this neurovascular coupling gain is kept constant, the parameter that is highly correlated with NBR amplitude will be κ. A disproportionate increase of this value leads to the ANC type of HRF. In this mechanism, the NBR can be seen as an exaggerated initial dip as a result of an abnormally enhanced O_2_ metabolism. It is hypothesized that this particular type of NBR may be clinically relevant while defining IZs. NBR can also have a pure vascular origin *via* an ABS effect. Here, we use a model proposed by Suarez et al. (39) that couples two Windkessels by a common artery to classify ABS types of HRFs. Simulations indicate that the parameter that determines the NBR amplitude is the resistance of the vessel (artery), relative to the total steady state resistance of the vasculature within the tissue, i.e., the arterioles, capillaries, and venules. A vascular anatomical network (VAN) model proposed earlier by Boas et al. (69) predicts also a relative decrease in CBF and O_2_ saturation around a brain area undergoing a positive functional hyperemic response. However, the authors are not aware of the application of the VAN model to study NBRs in epilepsy. The existence of ABS effect in the brain have been experimentally demonstrated by several groups (38, 41). Here, we recommend excluding IZs with ABS types of NBRs as potential candidates of SOZ. Some authors have suggested that a type of NBR might also result from the combination of blood backpressure and neuronal inhibition (47, 70). That would explain the presence of the poststimulus overshoot, which is in contrast to those observed in our pure vascular simulations. This undershoot is shown to be determined by the dynamics of the inhibitory neuronal state (44). Other causes including vein delayed compliance might also explain these transient. However, these last mechanisms were not investigated in this study.

### NBR Classification

In this article, we focus on the possibility of detecting and classifying the NBR mechanisms using the HRFs extracted from BOLD fMRI signals. We use linear models for the detection and estimation of the HRFs, i.e., GLM and NN-ARx methods, respectively. Under the assumption of the extended balloon model, the validity of the GLM was previously investigated by quantifying the effect size of second-order Volterra kernels (55). Under the same model, the validity of the NN-ARx to estimate HRFs was evaluated in (17, 45). Although linear models have been used to detect and reconstruct BOLD responses for decades, even before addressing the non-linear characteristics of BOLD signals (59, 71), we decided to analyze if linear models are able to accurately characterize PBRs and NBRs in epileptic patients. In general, for balloon/Windkessel models—with sporadic IED events and typical canonical-like responses (57), these linear models are suitable for spatial detection estimation of the HRF. We investigated if the NBR mechanisms can be solely classified from their BOLD responses. This is important for clinical applications when only standard fMRI paradigms are available or designed. Figure 3D shows that the machine learning classifier is able to differentiate PBR (red), ECI (yellow), NDA (green), ANC (cyan), and ABS (blue) in 100% of the cases. However, ECI and ABS were undistinguishable in some trials. In practice, this might be worse as there is a loss of sensitivity and specificity related to the usual misclassifications of IEDs, which is expert dependent. Furthermore, we cannot outline the possibility of having more than one NBR mechanism in the same area at the same time. Thus, failing to identify IZs with multiple NBR mechanisms could lead, in the worst-case scenario, to the incorrect clinical assessment. According to our hypothesis, an ABS/ECI misclassification will be the most critical case. However, these mechanisms are different in nature, and our model predicts different responses when using other imaging or recording modalities. At the expense of experimental feasibility, other imaging technique can be combined with our EEG-fMRI methodology to verify the predicted NBR mechanisms. For example, MION (38) and/or VASO (47) can be used to measure CBV concurrent with BOLD signals. Measurements of neuronal activity can be incorporated using ECoG/sEEG and EEG (27). In addition, CBF can be also included using arterial spin labeling (ASL) (30) and/or FAIR (47). In the case of ABS/ECI misclassification, a radiologist could use EEG concurrently recorded with ASL focused on the particular regions to verify whether there is a decrease in CBF. If no decrease in CBF is observed, ECI mechanisms should be expected. With these multimodal observations, we foresee a significant increase in the margins of classification of the NBR mechanisms, even in the case more than one is present in the same region (47, 72). It is important to highlight that none of the available imaging modalities (Table 1) provides conclusive results, and a thorough data evaluation in the surgical workup is needed for each clinical case. Our approach only aims at providing another level of EEG-fMRI data interpretation to improve the accuracy of this technique. To illustrate this, five specific clinical cases are discussed below.

### Epileptic Cases Discussion

We found an ABS type of NBR in patients 1 and 5. In patient 1, an NBR was located in the SPL and a PBR in the PG. The NBR was classified as ECI, which would occur *via* either U fibers or pure vascular phenomena. However, we cast doubt on ECI as we believe an inhibitory pathway from SPL to PG is rather weak. Note that the PG hosts the primary somatosensory cortex (S1) (73), which is a granular cortex that mainly receives somatotopic feedforward afferents from the ventral posterolateral and posteromedial relay nuclei (VPL and VPM) of the thalamus (74). In addition, the SPL, involved in transforming visual information in complex motor planning, has efferent pathways mainly to the premotor supplementary motor cortices in the precentral gyrus. A top–down inhibition from higher areas (prefrontal cortex) to the somatosensory area is mainly *via* efferent pathways. Moreover, both difussion spectrum imaging (DSI)-based connectivity (75) and cortical thickness–based connectivity (76) between SPL and PG are rather low. The mechanism might be ABS. Note that the detected BOLD responses are in the vascular domain of the middle central artery, at both sides of the postcentral sulcus. Thus, they might be sharing a final segment of the anterior parietal artery. Although we do not discard the existence of a venous blood backpressure effect, in which the regions could be sharing some anastomotic vein feeding the central sulcal vein or a branch of the superior anastomotic vein of Trolard, it has been reported that venous CBV changes are relevant only for longer stimuli (77). The NBR in patient 5 was found posterior to the cyst. We used data from this patient to illustrate the HRF estimation with the IRF method (SPM) and our NN-ARx method. Because of a probable revascularization around the cyst, angiography data from this patient will be required for a discussion about possible scenarios for the ABS effect. A frontal eye-field NBR with an ECI HRF was found in patient 2, which is most likely due to ictal propagation with frontal slow-wave responses. Slow-wave discharges have been found associated with NBR (13). DMN deactivations, like those found in patient 3, have been systematically reported in the literature for temporal lobe epilepsy (TLE) (11, 37) and for other types of focal epilepsy (36, 78). The pattern of deactivation depicted in Figure 6, with a predominance in the parietal node of the DMN ipsilateral to the focus, is similar to that reported ibyn Fahoum et al. (36) for four of five patients, with concomitant decrease of electrophysiological activity. Our results suggest that the PBR region might have afferents on the anterior part of the caudate nucleus that relay to central nodes of the DMN. This is consistent with the hypothesis of widespread secondary inhibition of non-seizing cortical regions *via* basal ganglia (79). The temporal profile of the PBR HRF in the right IFG experienced an unpredicted decay (or rebound) correlated with the amplitude of the NBR in the DMN nodes. This might be seen as an interruption of the PBR mechanism by inhibitory afferents coming from the regions exhibiting NDA, which in this case are present all around the right IFG. This might have implications in the interpretations of BOLD responses during IEDs or stimulation paradigms. If the location of the PBR is close to an affected RSN node, its HRF waveform might be misleading of the actual underlying PBR mechanism, due to either the interaction between mechanisms, i.e., inhibitory inputs from the NBR to the PBR region, or the effect of the BOLD spatial point-spread function. This rebound can be also explained as an increase in dHb due to an increase in neuronal activity in the NBR region, or even a vascular reallocation phenomena, as suggested by Hu and Huang (40). They observed positive and negative optical responses, concurrent with local field potentials (LFP) and multiunit activity (MUA) measurements, in rats during hindlimb electrical stimulation. Finally, it has been hypothesized that NDA is a disruption of RSN provoking a reduction of consciousness and cognitive reserve (36). Interestingly, our results suggest that a recovery from this disrupted state is not instantaneous. In our data, the estimated value for the recovery time constant was τ_*e*_2__ = 3*s*. The Epilepsy Connectome Project (ECP) (80) constitutes a huge database that contains clinical, neurophysiological, and resting-state fMRI (rs-fMRI) data of 105 patients with TLE and 55 healthy individuals as control. Using graph (nodes and edges) theory combined with rs-fMRI measures, a characterization of abnormal patterns in the local and global neuronal connectivity in TLE has been possible, thanks to the ECP. Our model-based method to identify different types of NBRs in epilepsy can help provide a neurophysiological foundation to the reported connectivity maps abnormalities. The ANC mechanism, as reported for patient 4, is related to a decrease in the CBF/CMRO_2_ balance. NBRs in the hippocampus of rats during bicuculline-induced generalized tonic–clonic seizures were associated with this type of mechanism (81). The authors reported that, even with higher LFP/MUA activity in the hippocampus, as compared to the cortex, the CBF was lower, and the CMRO_2_ was higher, yielding to NBRs in the hippocampus. Quantitatively, the unbalance corresponds to a decrease in the ratio $\frac{\varepsilon }{\kappa }$ in the OTT model (56), which is $\sim \frac{0.4}{0.05}=8$ for normal positive responses. Song et al. (35) estimated a ratio approximately five-fold smaller in rat with focal cortical seizures. Although the rCBF/CMRO_2_ coupling was reported to be preserved in human IEDs without any apparent lesion (82), we do not discard the possibility of an unbalance produced by a more critical state of tissue pathology. For example, the typical calcification of the surrounding blood vessels present in TSC tumors (83) could hamper the expected IED-induced increase of rCBF. We estimated κ = 0.51*s*^−1^ for the IED-related ANC mechanism around the lesion, which yields $\frac{0.28}{0.51}=0.55$, 14 times smaller than the normal values.

### Final Remarks

It is worth noting the foreseeable boost that BOLD modeling will have with the advent of new and optimized sequences in high-field spin-echo fMRI, with the considerably improved ability to measure high-resolution layer-dependent BOLD images and correlates of rCBV and rCBF (47, 70, 84–89). This allows for the construction and estimation of more detailed models of BOLD generation, through understanding of the actual role of arteries, capillaries, and veins in the generation of these observables and the possible biases that the variability of neurovascular/metabolic coupling, CBV, and signal-to-noise ratio (SNR) across layers could introduce. For example, it has been reported that the baseline CBV distribution varies over cortical layers biasing fMRI signal to layers with high CBV values (77). This affects the interpretation of what the contribution of the different vascular compartments to the average low-resolution BOLD response is.

## Data Availability Statement

The datasets presented in this article are not readily available because they are from clinical cases and this would jeopardize patient privacy. Requests to access the datasets should be directed to jrieradi@fiu.edu.

## Ethics Statement

The studies involving human participants were reviewed and approved by Western IRB, USA. Written informed consent to participate in this study was provided by the participants' legal guardian/next of kin.

## Author Contributions

AS: data analysis and model development. PV-H: data collecting and data analysis. BB: data analysis and clinic case discussion. CD: IED detection and classification. JB-B: data analysis. JR: research design, discussion, and writing. PV-H: data collecting, data analysis, and model development. All authors contributed to the article and approved the submitted version.

## Funding

This work was supported by the National Institutes of Health (R56NS094784-01A1).

## Conflict of Interest

The authors declare that the research was conducted in the absence of any commercial or financial relationships that could be construed as a potential conflict of interest.

## Publisher's Note

All claims expressed in this article are solely those of the authors and do not necessarily represent those of their affiliated organizations, or those of the publisher, the editors and the reviewers. Any product that may be evaluated in this article, or claim that may be made by its manufacturer, is not guaranteed or endorsed by the publisher.
