# Supplementary material for: Identification of Negative BOLD Responses in Epilepsy Using Windkessel Models
Source: Front Neurol. 2021 Oct 8;12:659081. doi: 10.3389/fneur.2021.659081 (PMC8531269; doi:10.3389/fneur.2021.659081)
Supplement: Supplementary file 1 [file Presentation_1.pdf]

Supplementary Material

Appendix - The biophysical model

The model can be written in the following general state-space form:

$$d\mathbf{x} = \mathbf{f}(\mathbf{x}, \boldsymbol{\theta}, u(t))dt + \text{diag}(\boldsymbol{\sigma}_x)d\mathbf{w}$$

$$y = h(\mathbf{x}, \boldsymbol{\theta}) + \zeta$$

where **x** is the column vector comprising the state variables (Option 1: $n = 22$; Option 2: $n = 20$) (**Table A2**), $\boldsymbol{\theta}$ is the vector of model parameters (**Table A3**), **f** is a vector with the drift functions (**Table A1**), $\boldsymbol{\sigma}_x$ is the vector with the variances of the state variables (the diffusion terms), h is the observation function (**Table A1**, Equation A4), $d\mathbf{w}$ is a Brownian stochastic process and $\zeta \sim N(0, \sigma)$ represents instrumental Gaussian noise. The system is driven by an input $u(t)$. All the equations (**Table A1**) and parameters (**Table A3**) taken from previous studies are respectively referenced, including those new proposed in this study. The biophysical model is based on two connected windkessel regions (1 and 2), as depicted in **Figure A1**.

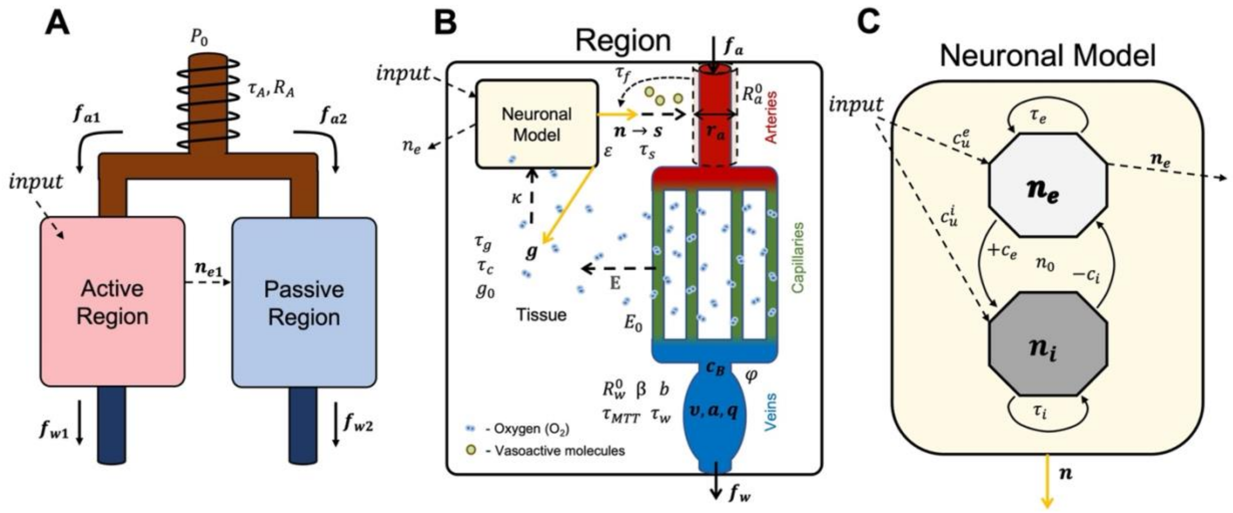

Figure A1. *Model of connected windkessels with viscoelastic compliance, two-state neuronal dynamics and tissue/blood O_2 dynamics. Bold symbols in red represent the state variables outlined in **Table A2**; symbols in black represent model parameters outlined in **Table A3**. A) Regions 1 and 2 are connected by a common supplying artery, with blood resistance and inertia. The whole system is under a common constant pressure. The external input acts on region 1. The latter influences the dynamics of region 2 by means of neuronal or vascular. B) Model details each region. The input acts on neuronal activity, which is represented by a yellow box. Neurovascular and neurometabolic couplings are represented with yellow arrows. That is, changes in the total neuronal activity, **n**, i.e., the weighted sum of excitatory and inhibitory contributions, causes the release of a vasoactive signal that is autoregulated by CBF (neurovascular coupling). O_2 is extracted by tissue and consumed by neurons. This extraction (OEF) depends on a set of state equations that model its dynamics, as well as those of tissue and blood O_2 concentration. Neuronal activity also modulates the $CMRO_2$ (neurometabolic coupling). These dynamics determine the amount of dHb in the windkessel. The windkessel volume is determined by the difference between the input and output CBF and viscoelastic properties. The BOLD signal is determined by the CBV and dHb, relative to their baseline values. C) Neuronal model. Two states—excitatory and inhibitory—are self- and influencing each other through effective connectivity. The input to region 1 is the external input, while the input to region 2 is the excitatory activity of region 1.*

Table A1. *Equations for the drift term of each region. The system is driven by an input $u(t)$. * - The P-DCM model (Havlicek et al., 2015) was extended to include an excitatory input into the inhibitory population ($c_{u_j}^{i}$). All the equations in this table are used for all, unless specified otherwise.*

Model type	Equation		Variable	Reference
	$\tau_{e_j}\dot{n}_{e_j} = 1 - n_{e_j} - c_{i_j}\left(n_{i_j} - 1\right) + c_{u_j}^{e} input_j$	(A1a)	Excitatory neuronal activity	
**Neuronal**	$\tau_{i_j}\dot{n}_{i_j} = 1 - n_{i_j} + c_{e_j}\left(n_{e_j} - 1\right) + c_{u_j}^{i} input_j$	(A1b)	Inhibitory neuronal activity	This study
	$input_1 = u(t), input_2 = n_{e_1} - 1, n_{e,i_j} \geq n_0$		Inputs	
	$n_j = f_e n_{e_j} + (1 - f_e) n_{i_j}$		Total neuronal activity	
	$\dot{s}_j = \varepsilon\left(n_j - 1\right) - \frac{s_j}{\tau_s} - \frac{f_{a_j} - 1}{\tau_f}$	(A2a)	Vasoactive signal	(Friston et al., 2000)
	Option 1: $\dot{r}_{a_j} = -r_{a_j}^2 s_j$	(A2b.1a)	Relative input resistance	
	$L_A \boldsymbol{M}\dot{\boldsymbol{f}}_{\boldsymbol{a}} = \mathbf{1} - R_A \boldsymbol{M}\boldsymbol{f}_{\boldsymbol{a}} - R_a^{(0)}\boldsymbol{R}_{\boldsymbol{a}}\boldsymbol{f}_{\boldsymbol{a}} - \left(1 - 2R_A + R_a^{(0)}\right)\boldsymbol{p}$	(A2b.1b)	Relative arteriole CBF	(Suarez et al., 2021)
	$\boldsymbol{R}_{\boldsymbol{a}} = \begin{pmatrix} r_{a_1} & 0 \\ 0 & r_{a_2} \end{pmatrix}, \boldsymbol{M} = \begin{pmatrix} 1 & 1 \\ 1 & 1 \end{pmatrix}$ and $\boldsymbol{f}_{\boldsymbol{a}} = \begin{pmatrix} f_{a_1} \\ f_{a_2} \end{pmatrix}, \boldsymbol{p} = \begin{pmatrix} v_1^{\beta}/a_1 \\ v_2^{\beta}/a_2 \end{pmatrix}$			
**Neurovascular coupling & Hemodynamic**	*Option 2:* $\dot{f}_{\boldsymbol{a}_j} = s_j$	(A2b.2a)	Relative arteriole CBF	(Friston et al., 2000)
	$\tau_{MTT}\dot{v}_j = f_{a_j} - f_{w_j}$	(A2c)	Relative CBV	
	$f_{w_j} = \frac{v_j^{\alpha+\beta}}{a_j}$		Relative windkessel CBF	(Zheng and Mayhew, 2009)
	$\tau_w \dot{a}_{\boldsymbol{j}} = -a_j + \exp\left(-b\dot{v}_j\right)$	(A2d)	Nonlinear viscoelastic	
	$\tau_{MTT}\dot{q}_j = \frac{E_j}{E_0} f_{a_j} - f_{w_j}\frac{v_j}{q_j}$	(A2e)	Relative dHb	(Buxton et al., 2004)
	ANC: $\frac{\varphi}{f_{a_j}}\dot{E}_j = -E_j + \left(1 - g_j\right)\left(1 - \left(1 - \frac{E_0}{1 - G_0}\right)^{1/f_{a_j}}\right)$	(A3a)		
**Neurometabolic coupling**	Steady state: $E_j = (1 - G_0)\left(1 - \left(1 - \frac{E_0}{1 - G_0}\right)^{1/f_{a_j}}\right)$		OEF	(Zheng et al., 2002)
	ANC: $\frac{\varphi}{f_{a_j}}\dot{c}_{B_j} = g_j - c_{B_j} - E_j/\ln\left(1 - \frac{E_j}{1 - g_j}\right)$	(A3b)	Blood O_2 concentration	
	ANC: $\tau_g \dot{g}_j = -\left(g_j - c_{B_j}\right)\ln\left(1 - \frac{E_0}{1 - G_0}\right) - E_0\left(\kappa n_j + 1\right)$	(A3c)	Tissue O_2 concentration	
**Observation**	$y_j = V_0\left[k_1\left(1 - q_j\right) + k_2\left(1 - \frac{q_j}{v_j}\right) + k_3\left(1 - v_j\right)\right]$	(A4)	BOLD	(Buxton et al., 1998)

Note: Subscripts j = {1, 2} denotes each jth windkessel region.

IED simulations

For the theoretical evaluation of the HRF, instantaneous pulses were used. However, actual IED are 70-200 ms; hence, the external input $u(t)$ was modeled as a single or a train of very short pulses. Such pulse can be viewed as a fast depolarization of the neurons, follow by a fast repolarization:

$$u(t) = u_0\left(e^{-t/t_r} - e^{-t/t_d}\right)$$

where $t_r = 5$ ms and $t_d = 2.5$ ms are the rise and decay times of the pulse, respectively, and u_0 is adjusted to provoke a maximum amplitude of 1% BOLD signal change when generating a typical PBR (Friston, 2002; Logothetis et al., 2001; Zheng et al., 2002).

Table A2. *States variables (and their steady state, baseline or initial values) of the biophysical models. A relative variable x is defined as the ratio of its value to its baseline, i.e., $X/X^{(0)}$. These state variables are defined for each region. We omit the j subscripts for brevity.*

State variable	Steady state /initial value/ baseline
Relative excitatory neuronal activity ($\boldsymbol{n_e}$)	1
Relative inhibitory neuronal activity ($\boldsymbol{n_i}$)	1
Vasoactive signal ($\boldsymbol{s}$)	0
Relative arteriole resistance ($\boldsymbol{r_a}$)	1
Relative cerebral blood volume ($\boldsymbol{v}$)	1
Relative cerebral blood flow in the arteriole ($\boldsymbol{f_a}$)	1
Relative pressure to volume relation constant ($\boldsymbol{a}$)	1
Relative amount of deoxy-hemoglobin in the balloon/windkessel compartment ($\boldsymbol{q}$)	1
Oxygen extraction fraction (OEF) ($\boldsymbol{E}$)	E_0
Tissue O_2 concentration normalized by plasma O_2 concentration in the arterial end of the capillary ($\boldsymbol{g}$)	G_0
Total blood O_2 concentration normalized by blood O_2 concentration in the arterial end of the capillary ($\boldsymbol{c_B}$)	$G_0 - E_0/ln(1 - E_0(1 - G_0)^{-1})$

Particular cases of the model

According to simulations, for the jth region, the balance between c_{i_j} and c_{e_j}, and between $c_{i_j}^u$ and $c_{e_j}^u$ in equations (A1a, A1b) determines the polarity of the BOLD response. Irrespective of the value of c_{i_j}, a PBR is obtained when $c_{e_1}^u = 1$ and $c_{i_j}^u = 0$; while an inhibitory-related NBR is obtained for $c_{i_j} = 2$ and $c_{i_j}^u = 2$. We use this value of c_{i_j} for all mechanisms since it is reasonable to assume that inhibitory gains are stronger than excitatory ones. Inhibitory interneurons tend to have synapses closer to the soma (Megías et al., 2001; Villa and Nedivi, 2016). We used the PBR configuration in region 1, i.e., $c_{i_1} = 2$ and $c_{i_1}^u = 0$, to simulate non-inhibition-related NBR mechanism, i.e., ANC and ABS. To simulate the ECI, we assume the input is acting on both states of region 1, i.e., $c_{e_1}^u = 1$, $c_{i_1}^u = 2$, while this effect is not transmitted to region 2, i.e., $c_{i_2}^u = c_{e_2}^u = 0$. In order to model the NDA mechanism, we set the parameter of the strength into the excitatory ($c_{e_2}^u = 1$) and inhibitory ($c_{i_2}^u = 2$) stages in region 2 (NBR) to their maximum values.

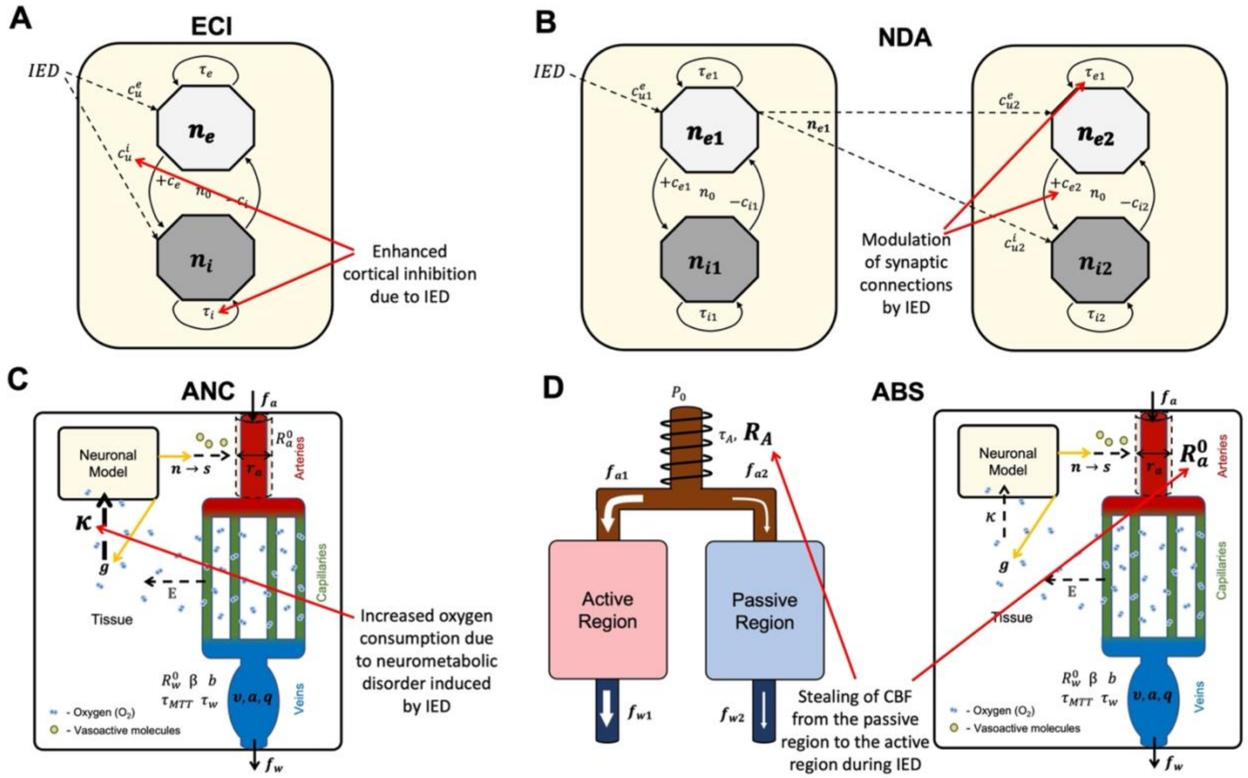

Figure A2. *Model configurations for the four different mechanisms. A) Neuronal configuration for ECI mechanism, where the IEDs enhance cortical inhibition. B) Neuronal configuration for NDA mechanism, where the synaptic connections are modulated by the IED. C) Neurometabolic imbalance due to altered consumption rate of oxygen during IED. D) ABS mainly depends on the resistance and of the feeding artery and the baseline resistance of both activated and passive regions.*

Except for NDA, times constants for the neuronal states are set to $\tau_{e_j} = 10\text{ms}$, to obtain a fast-neuronal response (like an IED) when an ultra-short input pulse is applied, and $\tau_{i_j} = 30\text{ms}$ to simulate a slower inhibitory-related recovery of the neuronal activity (similar to the hyperpolarizing wave seen after IEDs). These time constants are in the range of those typically used in neural mass modelling literature involving excitatory and inhibitory populations of neurons (David et al., 2006; Robinson, 2005). For ECI, τ_{i_1} can be as high as 200ms to simulate the slower recovery wave reported after IEDs, c.f. (Pittau et al., 2013). Varying this parameter within the interval [30ms, 200ms], we obtain respective variations in the NBR. Also, we assume that, due to conflicting interactions between the different nodes of the affected network, the recovery of the neuronal activity in NDA is much slower than in other mechanisms, i.e., $\tau_{e_2} = [3\text{s}, 10\text{s}]$, which defines its characteristic shape.

Table A3. *Parameters of the biophysical models. The assumed fixed values are those used in simulations performed to investigate the detectability of the NBR using GLM. In the estimation of the parameters, the values are allowed to vary within the provided ranges. These are also the ranges from which the parameters are randomly sampled to generate the ensemble of HRFs used to build the machine learning classifier. Note that these ranges are not the most general ranges, but those leading to NBR mechanisms. The values in this table are used for all models, unless specified otherwise. There is no need to provide uncertainty to all parameters, only a subset is enough to provide the entire possible span of variability in the HRFs*.

Parameter (units)	Symbol	Assumed fixed value	Range of possible values	References
Neurovascular coupling gain (input efficacy)	ε	0.28	[0.2, 0.5]	(Friston. 2002)
Time constant of the vasoactive signal (s)	τ_s	1.1	[0.9, 1.2]	
Autoregulation time constant (s)	τ_f	1.6	[1.4, 2.2]	
Total tissue mean transit time (s)	τ_{MTT}	1.8	[1.2, 2.4]	(Friston, 2002; Havlicek et al., 2015)
Diminished reserved volume exponent	β	2	—	(Boas et al., 2008)
Exponent of Poiseuille's law	α	2	—	(Zheng and Mayhew, 2009)
Steady state oxygen extraction fraction	E_0	0.5	—	(Friston. 2002)
Time constant of $\boldsymbol{a}$ (s)	τ_w	6.68	—	(Zheng and Mayhew, 2009)
Gain modeling the influence of the variation of CBV on $\boldsymbol{a}$ (s)	b	7.6	—	
Minimum allowed value of $\boldsymbol{n}$ (s)	n_0	0.4	—	(Logothetis et al., 2001)
Neuronal excitation time constant in region 1 (ms)	τ_{e_1}	10	—	Section below of particular cases
Neuronal excitation time constant in region 2 (ms)	τ_{e_2}	10<br>NDA: 3000	NDA:<br>[1000, 10^4]	
Neuronal inhibitory time constant in region 1 (ms)	τ_{i_1}	30	—	
Neuronal inhibitory time constant in region 2 (ms)	τ_{i_2}	30	ECI: [30, 200]	
Excitatory → inhibitory connectivity in region 1	c_{e_1}	1	—	
Excitatory → inhibitory connectivity in region 2	c_{e_2}	1<br>NDA:0.01	—	
Inhibitory → excitatory connectivity in region 1	c_{i_1}	2	—	
Inhibitory → excitatory connectivity in region 2	c_{i_2}	2		
Input strength to excitatory state in region 1	$c^e_{u_1}$	1	—	
Input strength to excitatory state in region 2	$c^e_{u_2}$	1	—	
Input strength to inhibitory state in region 1	$c^i_{u_1}$	0<br>ECI: 2	—	
Input strength to inhibitory state in region 2	$c^i_{u_2}$	0<br>NDA: 2	—	
Fraction of excitatory neurons	f_e	0.75	—	
Arterial inertial time constant (ms)	τ_A	1	—	(Suarez et al., 2021)
Feeding artery resistance	R_A	0<br>ABS: 0.17	ABS:<br>[0.13, 0.27]	
Baseline arteriole resistance:	R^0_a	0.39	—	
Neuro-$CMRO_2$ coupling gain	κ	0.05<br>ANC: 0.35	ANC:<br>[0.15, 0.55]	(Song et al., 2016; Zheng et al., 2002)
Parameter shaping the dynamics of $\boldsymbol{E}$ and $\boldsymbol{C_B}$ (s)	φ	0.15	—	(Zheng et al., 2002)
Steady state value of $\boldsymbol{g}$	G_0	0.1	—	
Time constant of $\boldsymbol{g}$ (s)	τ_g	1.2	[0.12, 2.4]	
Resting blood volume fraction	V_0	0.02	—	(Friston et al., 2000)
Constant in the observation equation	k_1	$4.3\vartheta_0 E_0 T_E$	—	(Havlicek et al., 2015)
Constant in the observation equation	k_2	$\epsilon r_0 E_0 T_E$	—	
Constant in the observation equation	k_3	$1-\epsilon$	—	
Ratio of intra-vascular to extra-vascular fMRI signal contributions (s^{-1})	ϑ_0	40.3	—	
Frequency offset at the surface of a blood vessel for fully deoxygenated blood for Gradient Echo (GE) sequence at 1.5T (s^{-1})	ϵ	1.32	—	
Sensitivity of changes in intra-vascular signal relaxation rate with changes in oxygen saturation for GE sequence at 1.5T	r_0	15	—	
Echo time (ms)	T_E	45	—	Experiments in "EEG-fMRI data" with a GE sequence at 1.5T

For all mechanisms, we force the neuronal activity of both states to be higher than 40% ($n_0 = 0.4$) of its baseline activity. Contribution of excitatory (inhibitory) activity to the generation of the BOLB response is set to 75% (25%), following the proportion of excitatory/inhibitory neurons in the human cortex.

Under normal conditions, the neurometabolic coupling gain can be set to $\kappa = 0.05$ and the effect of the input on g, c_B and E is very little. For simplicity, we assume the steady state of equation A3a (**Table A1**), with $g = G_0$, to simulate and estimate the model for four particular types: ECI, NDA, ABS, and PBR.

Note that ANC and ECI do not need two interacting regions to happen, so we can disconnect them and consider a model with only one region. ABS is the only mechanism that is modeled as if the two regions were connected by the same feeding artery including the solenoid. For all others (ECI, NDA, ANC) we disconnect the windkessels from the common supplying artery. This corresponds to vessels with very large diameters or regions far apart from each other, i.e., independent blood flow supply. Although both resistances, $R_a^{(0)}$and R_A, are determinant on the stealing, the one that plays the biggest role on the negative part is R_A, (Suarez et al., 2021). The range of values for this parameter was set according to a range of stealing percent consistent to reported data (see **Table A3**).

References

Boas DA, Jones SR, Devor A, Huppert TJ, Dale AM. A vascular anatomical network model of the spatio-temporal response to brain activation. *Neuroimage.* (2008) 40:1116–29. doi: 10.1016/j.neuroimage.2007.12.061

Buxton RB, Wong EC, Frank LR. Dynamics of blood flow and oxygenation changes during brain activation: the Balloon model. MRM. (1998) 39:855–64. doi: 10.1002/mrm.1910390602

Buxton RB, Uludag K, Dubowitz DJ, Liu TT. Modeling the hemodynamic response to brain activation. Neuroimage. (2004) 23:220–33. doi: 10.1016/j.neuroimage.2004.07.013

David, O., Kiebel, S.J., Harrison, L.M., Mattout, J., Kilner, J.M., Friston, K.J.,. Dynamic causal modeling of evoked responses in EEG and MEG. *Neuroimage* (2006) 30:1255-72. doi:10.1016/j.neuroimage.2005.10.045

Friston KJJ, Mechelli A, Turner R, Price CJJ. Nonlinear responses in fMRI: the Balloon model,

Volterra kernels, and other hemodynamics. *Neuroimage.* (2000) 12:466–77. doi: 10.1006/nimg.2000.0630

Friston KJJ. Bayesian estimation of dynamical systems: an application to fMRI. *Neuroimage.* (2002) 16:513–30. doi: 10.1006/nimg.2001.1044x

Havlicek M, Roebroeck A, Friston K, Gardumi A, Ivanov D, Uludag K. Physiologically informed dynamic causal modeling of fMRI data. *Neuroimage.* (2015) 122:355–72. doi: 10.1016/j.neuroimage.2015.07.078

Logothetis NK, Pauls J, Augath M, Trinath T, Oeltermann A. Neurophysiological investigation of the basis of the fMRI signal. *Nature* (2001) 412:150-57. doi:10.1038/35084005

Pittau F, Fahoum F, Zelmann R, Dubeau F, Gotman J. Negative BOLD response to interictal epileptic discharges in focal epilepsy. Brain Topogr. (2013) 26:627–40. doi: 10.1007/s10548-013-0302-1

Megías M, Emri Z, Freund T, Gulyás A. Total number and distribution of inhibitory and excitatory synapses on hippocampal CA1 pyramidal cells. *Neuroscience* (2001) 102:527-40. doi:10.1016/S0306-4522(00)00496-6

Robinson P. Neural field theory of synaptic plasticity. *J. Theor. Biol.* (2011) 285:156–63. doi:10.1016/j.jtbi.2011.06.023

Song Y, Torres R, Garcia S, Frometa Y, Bae J, Deshmukh A, et al. Dysfunction of neuro-vascular/metabolic coupling in chronic focal epilepsy. *IEEE Trans Biomed Eng.* (2016) 63:97–110. doi: 10.1109/TBME.2015.2461496

Suarez A, Valdes-Hernandez PA, Moshkforoush A, Tsoukias N, Riera J. Arterial blood stealing as a mechanism of negative BOLD response: from the Steady-flow with nonlinear phase separation to a Windkessel-based model. *J Theor Biol.* (2021) 529:110856. doi: 10.1016/j.jtbi.2021.110856

Villa, K.L., Nedivi, E., 2016. Excitatory and Inhibitory Synaptic Placement and Functional Implications, in: Dendrites. Springer Japan, Tokyo, pp. 467–487. doi:10.1007/978-4-431-56050-0_18

Zheng Y, Martindale J, Johnston D, JonesM, Berwick J,Mayhew J. A model of the hemodynamic

response and oxygen delivery to brain. *Neuroimage.* (2002) 16:617–37. doi: 10.1006/nimg.2002.1078

Zheng Y, Mayhew J. A time-invariant visco-elastic Windkessel model relating blood flow and blood volume. *Neuroimage.* (2009) 47:1371–80. doi: 10.1016/j.neuroimage.2009.04.022
